# Supplementary material for: Molecular transport through large-diameter DNA nanopores
Source: Nat Commun. 2016 Sep 23;7:12787. doi: 10.1038/ncomms12787 (PMC5036142; doi:10.1038/ncomms12787)
Supplement: Supplementary Information — Supplementary Figures 1-24, Supplementary Table 1, Supplementary Notes 1-5 and Supplementary References. [file ncomms12787-s1.pdf]

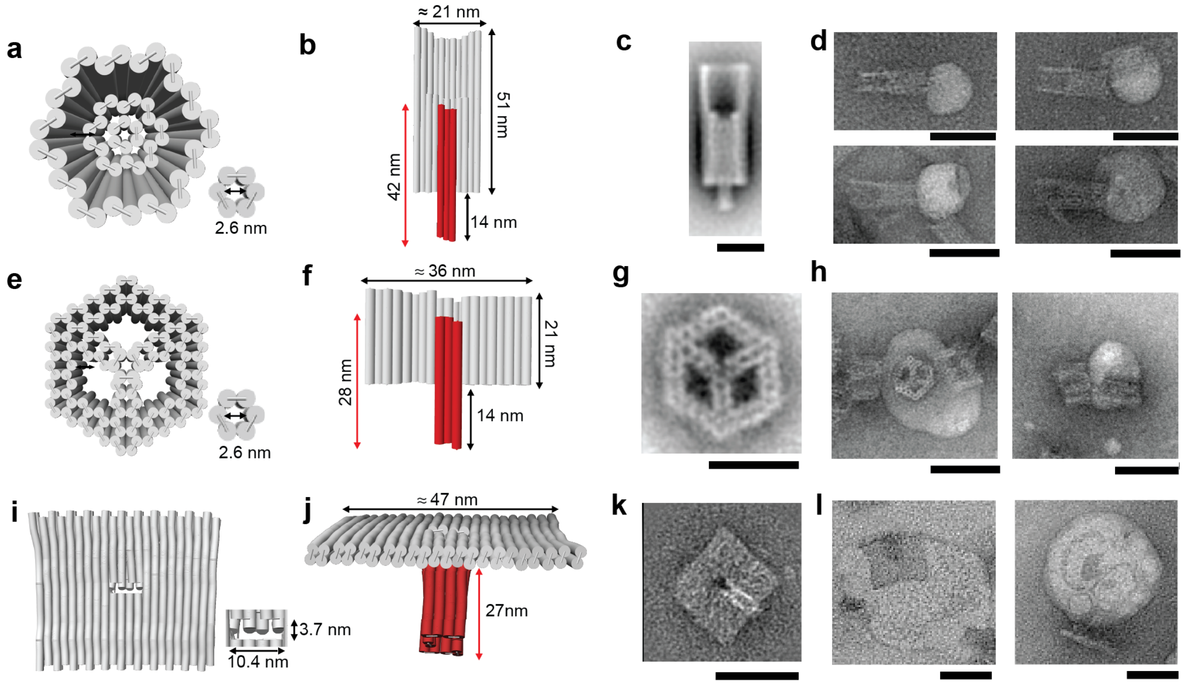

Supplementary Figure 1. Design and TEM imaging of all three pores. a, Top view of the pin pore showing a 2 nm pore b, Side view of the pin pore with dimensions of the vestibule. The transmembrane region is in red. c, Class average TEM image of the pin pore. d, TEM images of membrane interaction between the tocopherol modified pin pore and small unilamellar vesicles (SUVs). e, Schematic of the wheel pore which has an extended membrane interacting surface f, Side view of the wheel pore stem. g, TEM class average structure of the wheel pore h, TEM images of hydrophobically modified wheel pore with SUVs. i, Schematic of the T pore having a pore size of approximately 4 nm. j, Side view of the T pore. k, TEM class averaged image of the T pore. l, SUV interaction with tocopherol modified T pore. Scale bars: 50 nm.

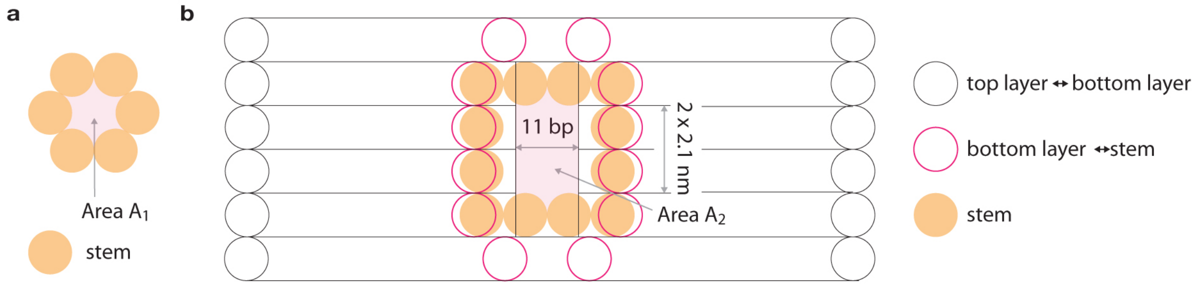

Supplementary Figure 2. Schema of the cross-sectional channel area for a, the pin and wheel pore and b, the T pore

a

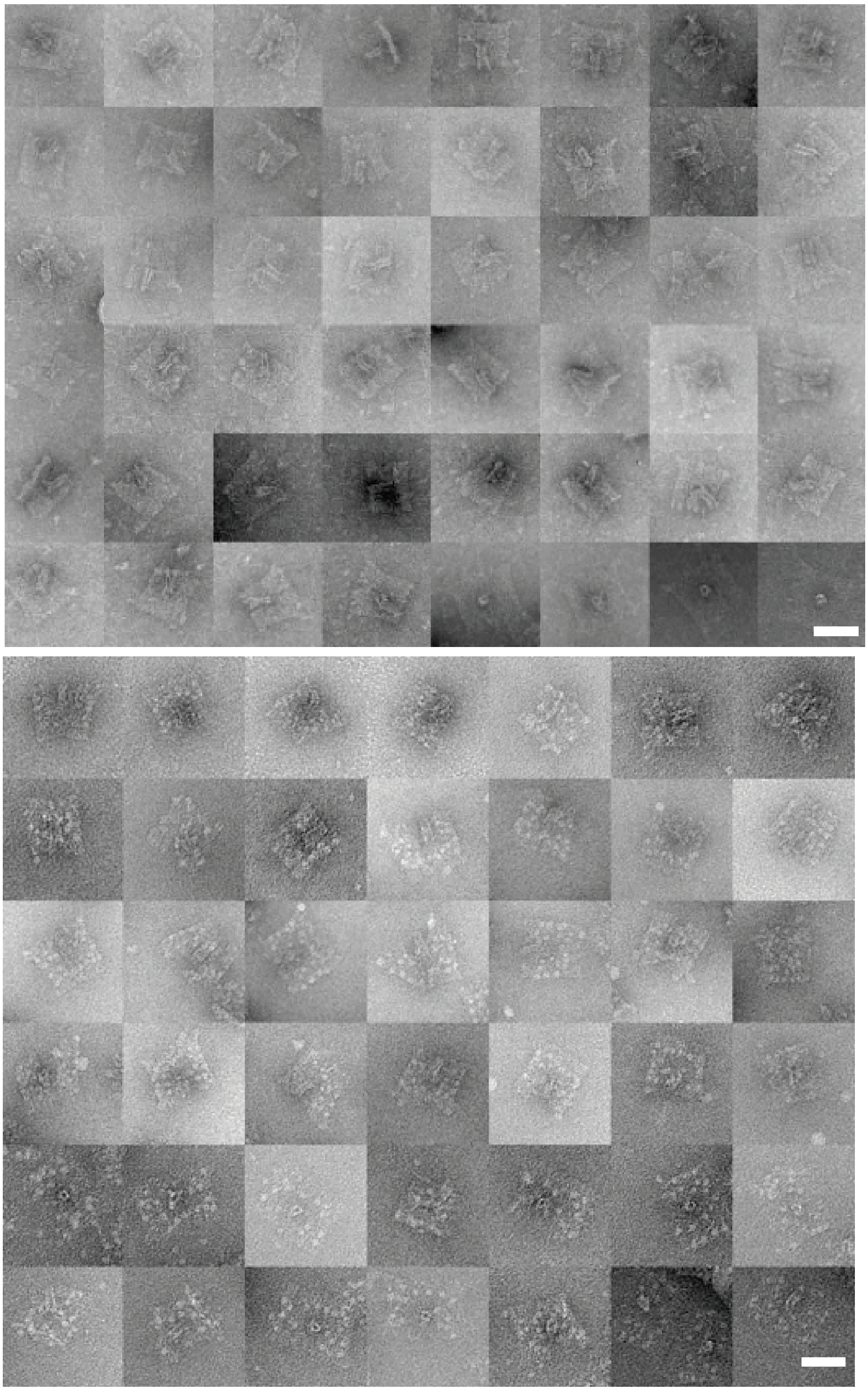

Supplementary Figure 3. a, TEM images of unmodified, filter-purified T pores. b, TEM images of the streptavidin modified T pore. Both the unmodified and the modified structures have similar shape and structure, indicating that the addition of streptavidin does not considerably distort the pore structure. Scale bar: 50 nm.

a

b

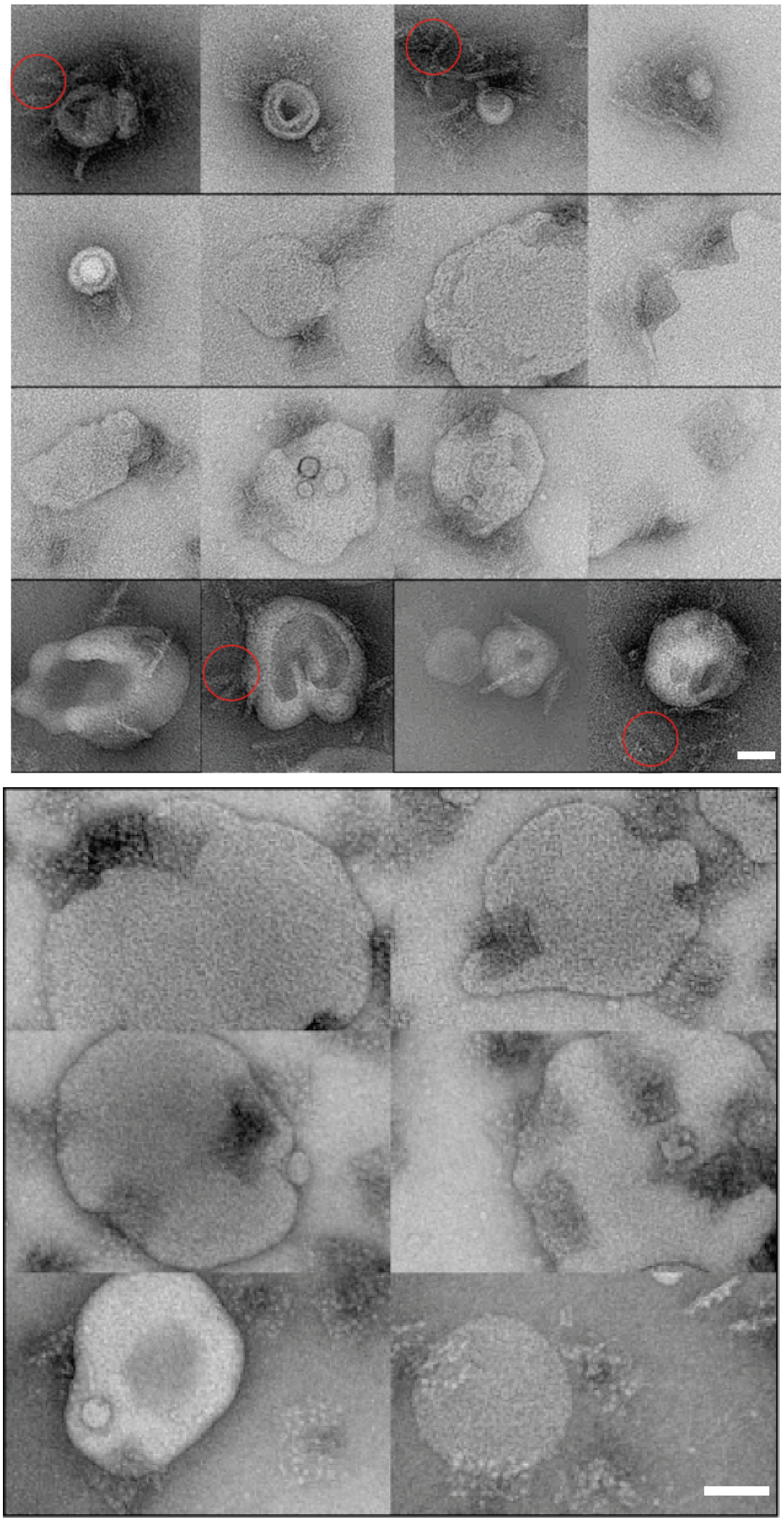

Supplementary Figure 4. TEM images of T pores interacting with SUVs. a, T pores modified with tocopherol. Aggregated pores are marked with red circles. b, T pores modified with streptavidin interacting with biotinylated SUVs. Note the lower degree of aggregation, which may provide an explanation for differences in the kinetics of the dye influx assays. Scale bar: 50 nm.

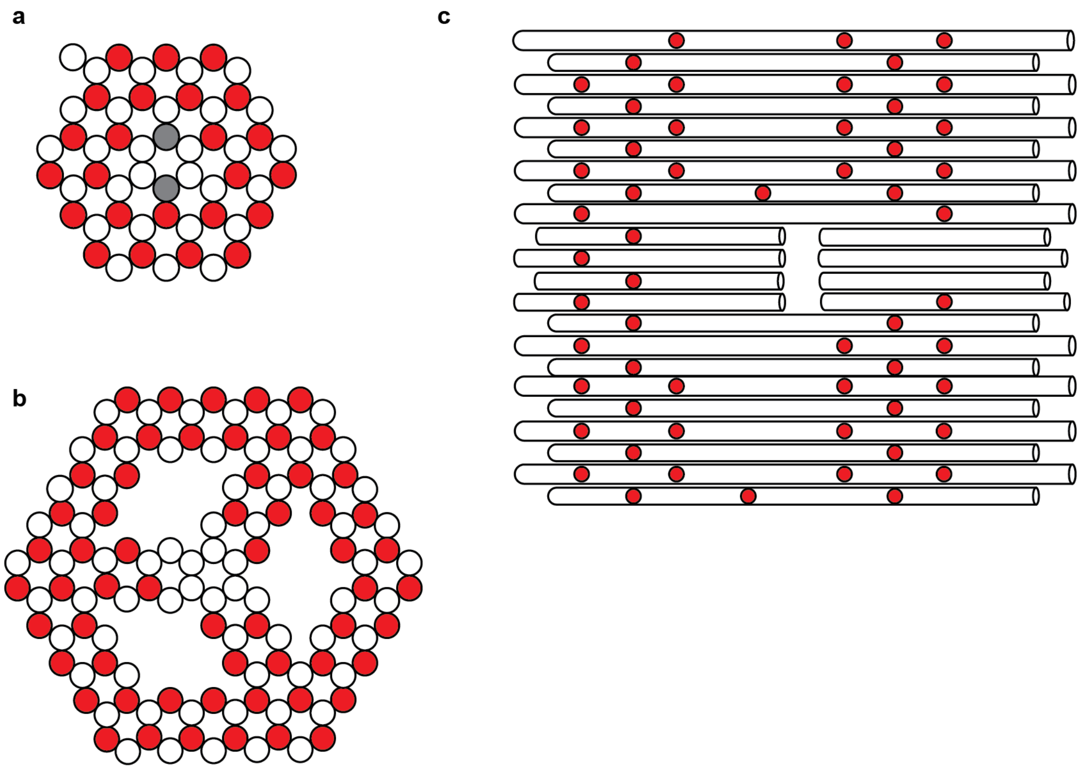

Supplementary Figure 5. Distribution of adaptors on the DNA nanostructures. a, The pin pore has 26 possible handle positions 24 marked in red and 2 in grey. Red circles in the schemes indicate modifications at the bottom of the DNA channel ‘barrel’ domain, gray circles are modifications protruding from the sides of the six helix stem. The handles are single-stranded staple extensions (all having the same sequence, 5’ TAACAGGATTAGCAGAGCGAGG 3’) to which a complementary adaptor strand having a tocopherol or biotin modification at its 5´end can hybridize (5’ CCTCGCTCTGCTAATCCTGTTA 3’). After modification, the resulting structure has 26 tocopherol or biotin moieties attached at the end of a now double stranded DNA. The positions are distributed across the structure to avoid tocopherol interactions between neighbouring anchors. b, The wheel pore has 57 modification positions at the bottom of its cap, which improves membrane incorporation, but also increases the tendency to aggregate. c, The T pore also has 57 modification positions as indicated in red.

a b

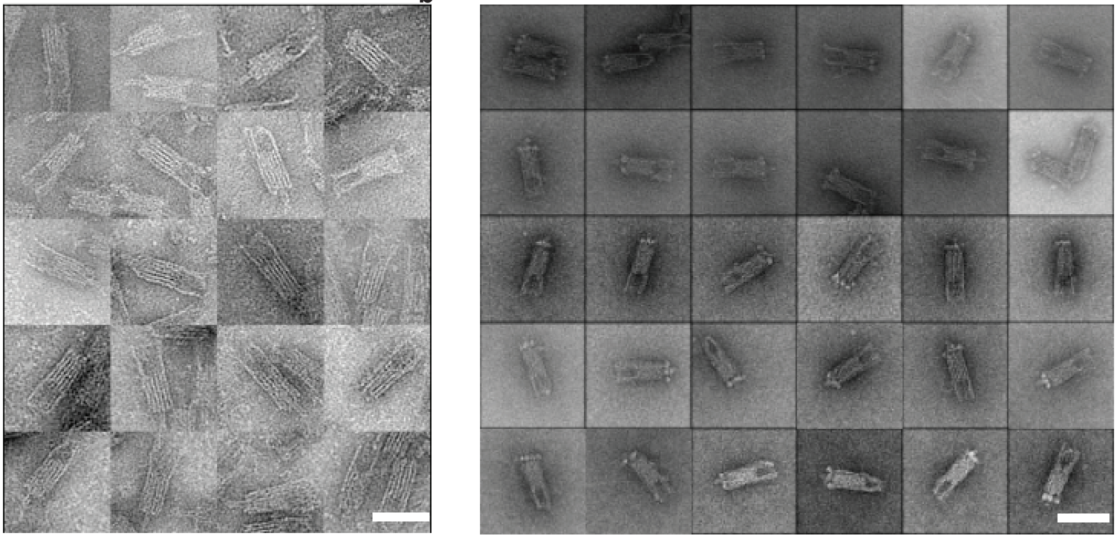

Supplementary Figure 6. TEM images of the pin pore. a, Unmodified pin pores. b, Biotinylated pin pores functionalized with streptavidin molecules. The streptavidin molecules are observed as white spots at the base of the pore where the biotinylated adaptors are present. Scale bars: 50 nm.

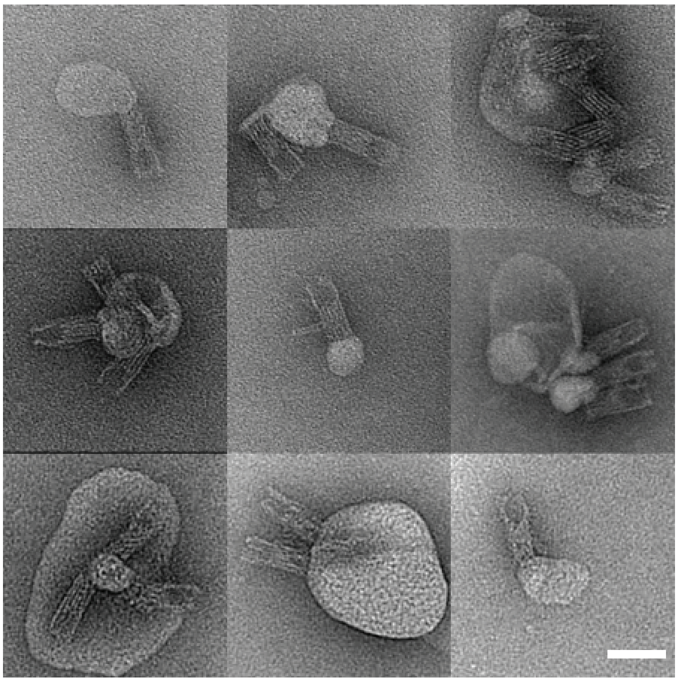

Supplementary Figure 7. TEM images of the pin pore interacting with small unilamellar vesicles (POPC). The pores are oriented perpendicularly to the membrane surface, which is the desired orientation for transmembrane pore formation. The pores exhibit some aggregation but incorporate well into SUVs, potentially also supported by the high curvature of the SUV membrane. Scale bar: 50 nm.

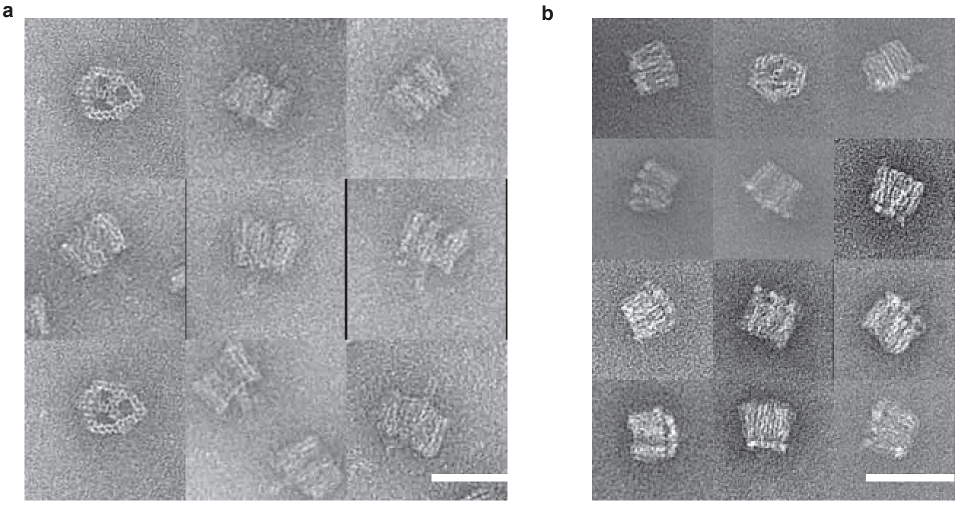

Supplementary Figure 8. a, TEM images of the wheel pore b, TEM images of the wheel pore modified with streptavidin molecules. The streptavidin molecules are observed as white spots at the base of the pore where the biotinylated adaptors are positioned. Scale bar: 50 nm.

.

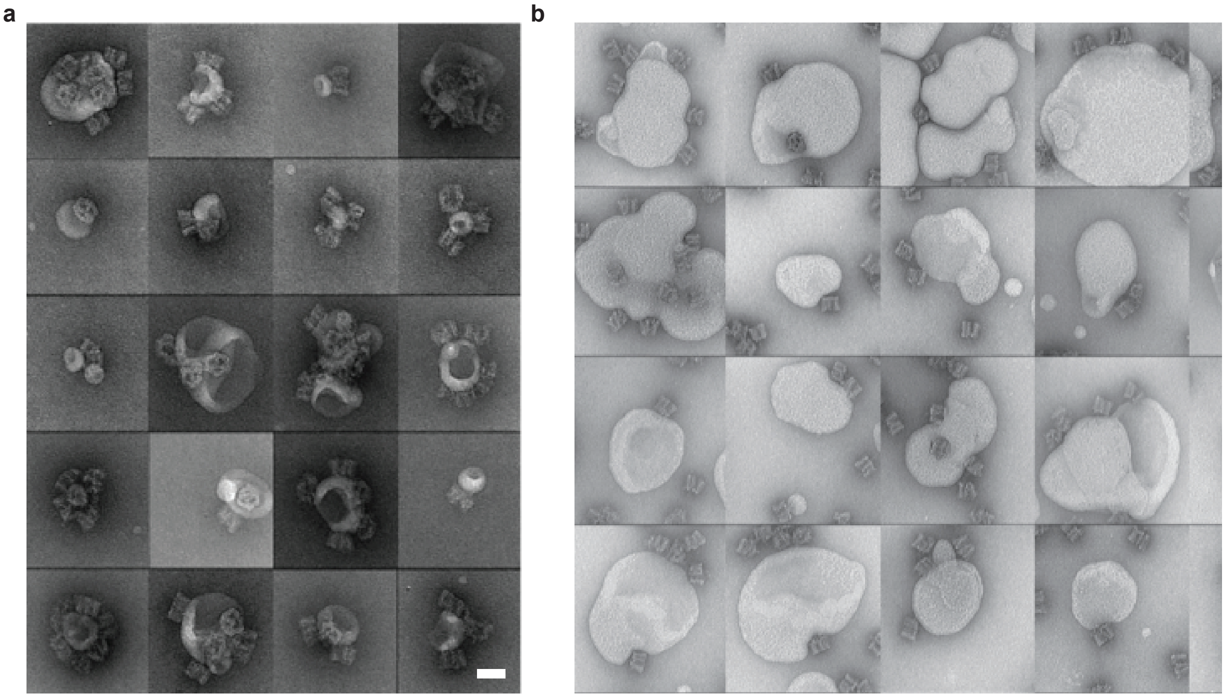

Supplementary Figure 9. a, TEM images of the wheel pore modified with tocopherol moieties interacting with SUVs. Note the presence of some aggregation of pores on the SUVs. b, TEM images of the wheel pore modified with streptavidin molecules interacting with SUVs containing 10% biotinylated lipids. The pores interacting with the SUVs are fewer in number than in the case of tocopherol-modified pores, but they are well eparated and no aggregates are observed. Some pores are present that are not interacting with the vesicles. Scale bar: 50 nm.

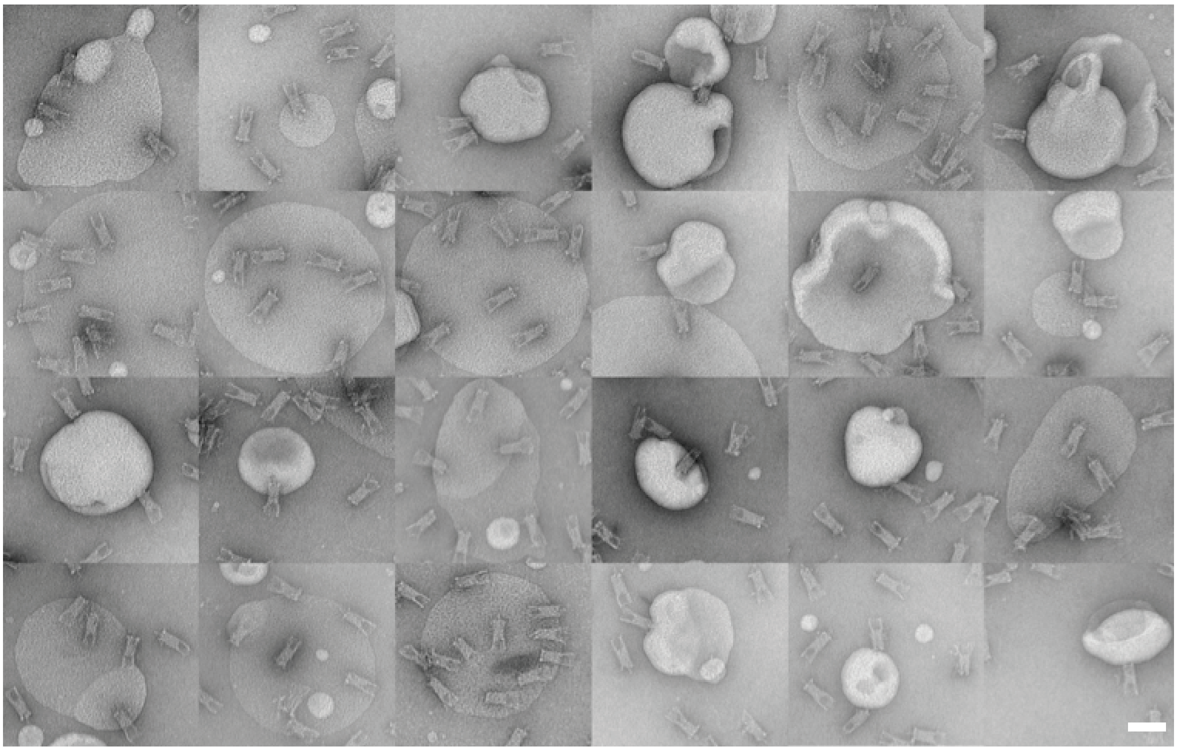

Supplementary Figure 10. a, Streptavidin modified pin pore interacting with SUVs containing biotinylated lipids. The overall shape of the pore structure is maintained after modification with the protein. Scale bar: 50 nm.

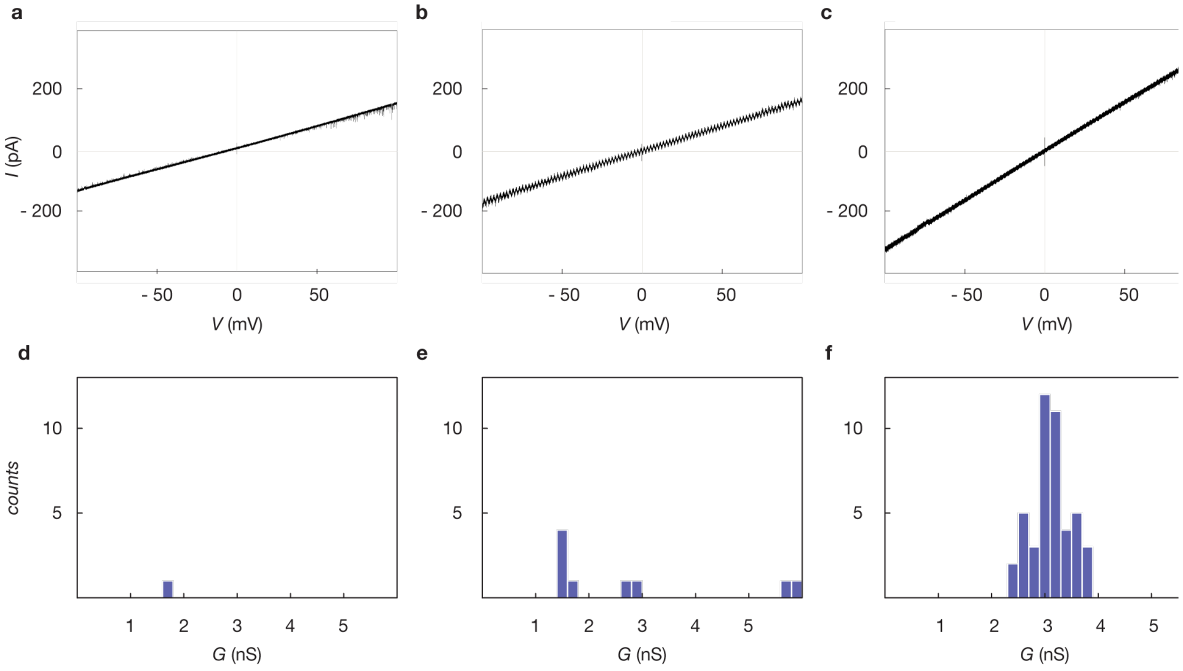

Supplementary Figure 11. I-V curve for a, the pin, b, the wheel and c, the T pore. Measured conductance values for d, the pin, e, the wheel and f, the T-pore.

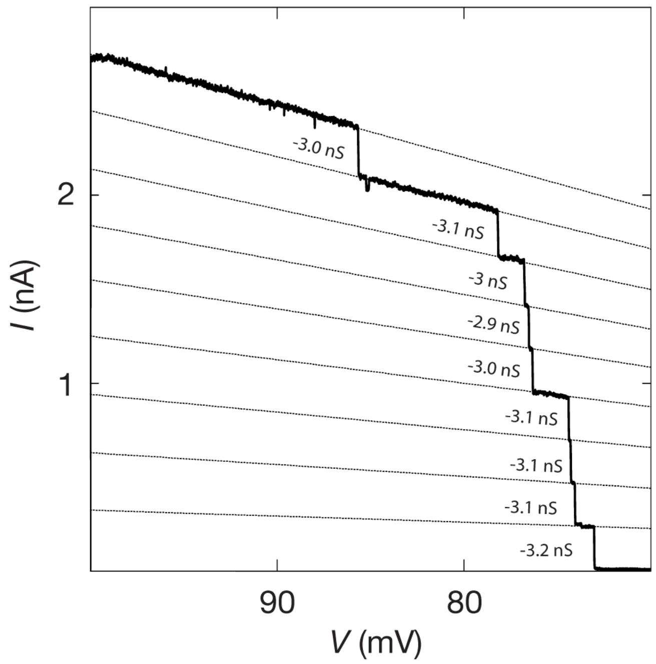

Supplementary Figure 12. In this experiment, incorporation of multiple T pores into a DIB membrane resulted in a conductance value of $G = 27$ nS. While the two droplets were separated from each other, a voltage ramp of 1 V/s, starting at + 100 mV was applied. The separation of the droplets leads to a splitting of the bilayer back into two monolayers. While separating the droplets, nine single current steps occurred. Dashed lines are a guide to the eye, indicating the contribution of the single pores.

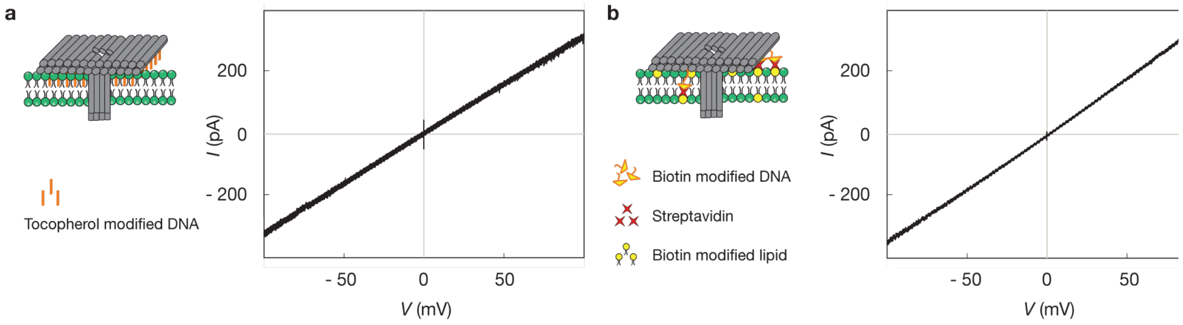

Supplementary Figure 13. I-V curve of the T-pore utilizing a, tocopherol or b, biotin-streptavidin for anchoring. Biotin-streptavidin anchoring results in a conductance in the same range as for the tocopherol modified pores.

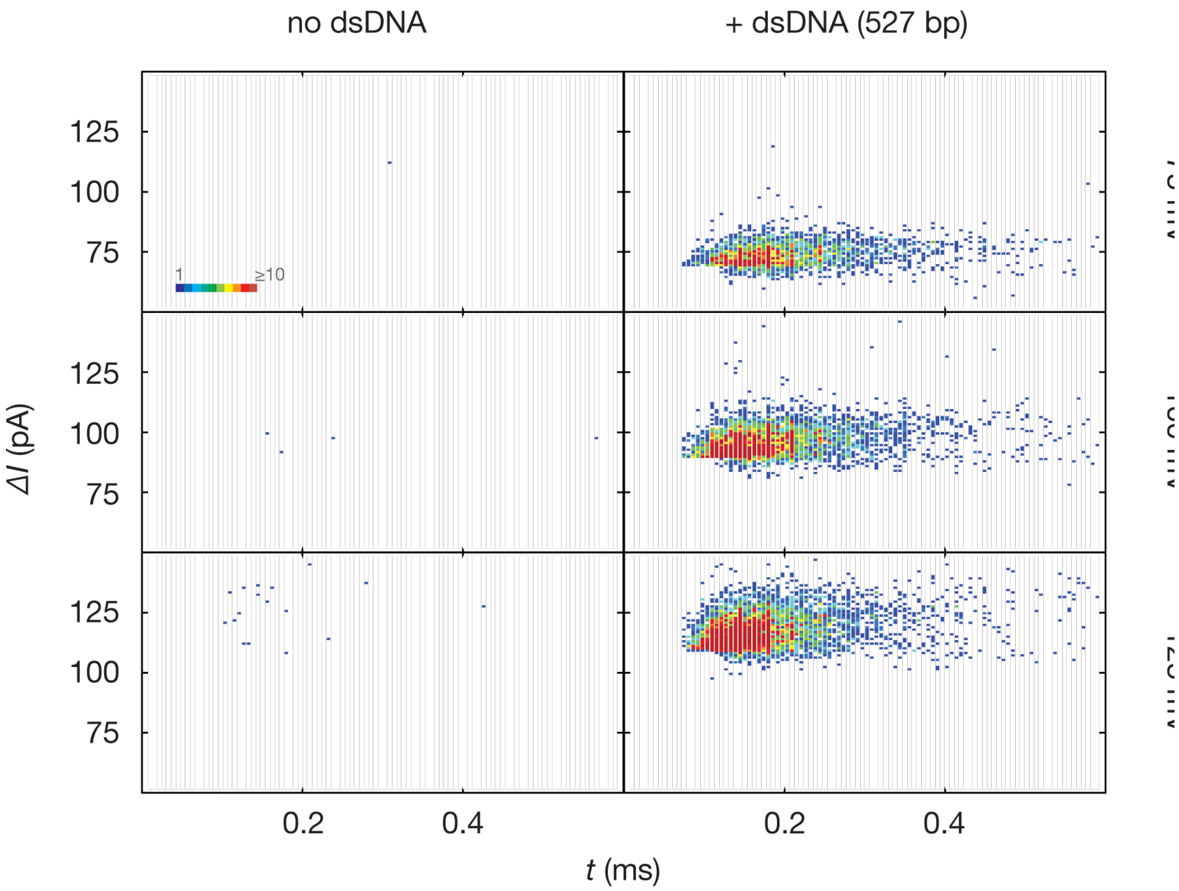

Supplementary Figure 14. Translocation experiments with the T pore at the droplet interface bilayer setup, using 527 bp long dsDNA analyte molecules. Recordings were performed before and after the addition of dsDNA at 75, 100 and 125 mV for 300 and 900 seconds respectively. The number of translocation events is represented by a heat plot.

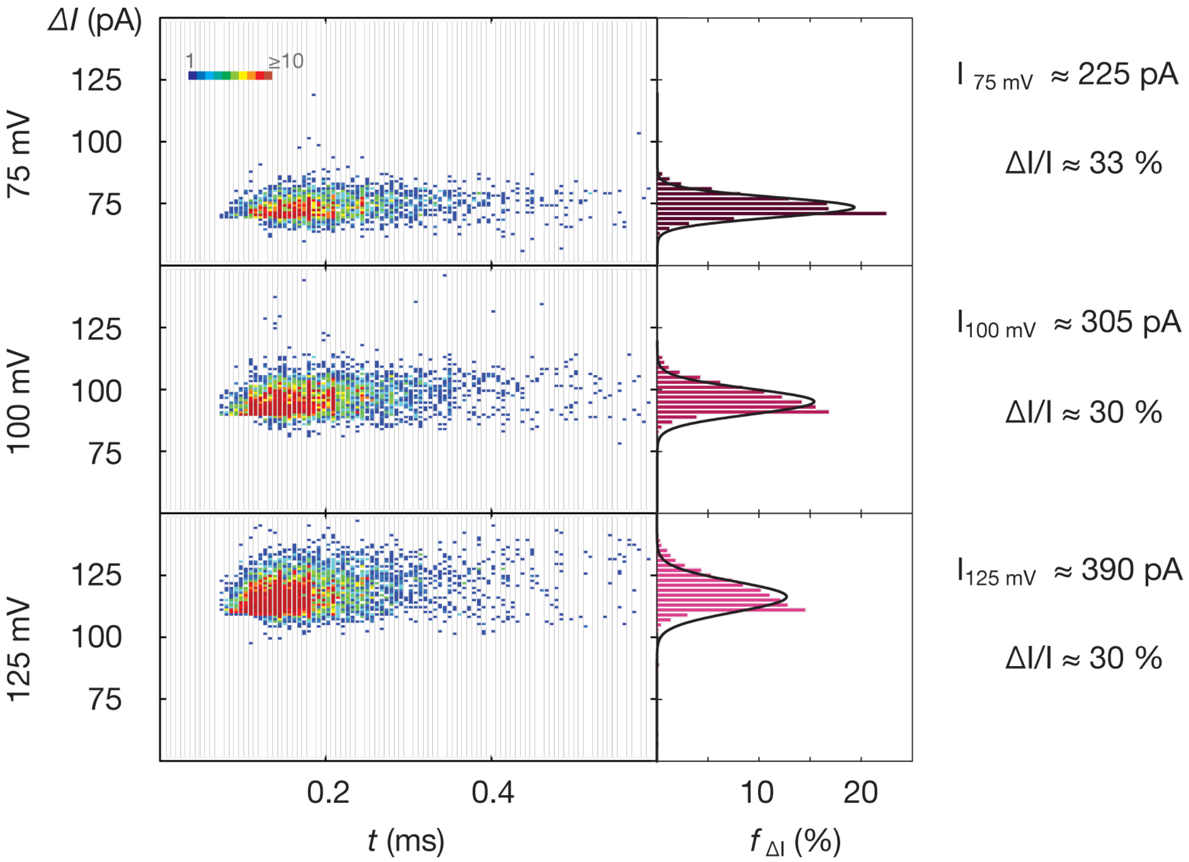

Supplementary Figure 15. Frequency count of ΔI, fit with a Gaussian distribution. The maximum of the Gaussian distribution determines the most probable ΔI and thereby $\Delta I/I$.

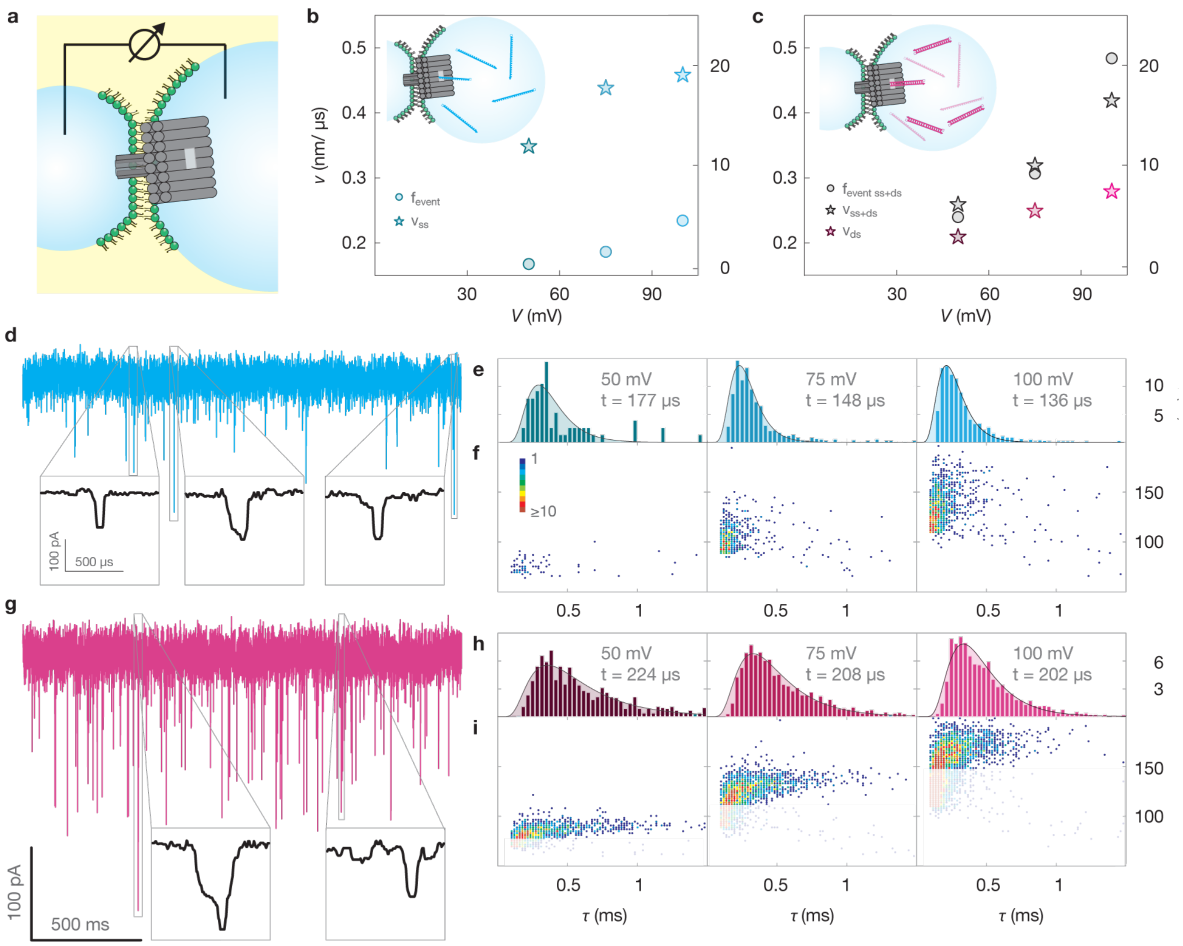

Supplementary Figure 16. Translocation experiments with the T pore at the droplet interface bilayer (DIB) setup. a, Schematic drawing of the DIB setup, b, event frequency and translocation velocities for ssDNA at 2M KCl and 5mM MgCl2, c, event frequency and translocation velocities for a mixture of 140 nt long ssDNA and 115 bp dsDNA and extracted event frequency and translocation velocities for dsDNA only. d, Example trace with ssDNA at 100mV. e, Distribution of the event length for ssDNA at 50, 75 and 100mV and f, all detected events for ssDNA at 50, 75 and 100mV. g, Example trace with ssDNA and dsDNA at 100mV. h, Distribution of the event length of the non-faded population at 50, 75 and 100mV and i, all detected events at 50, 75 and 100mV. All histograms were fitted with 1D drift diffusion regression analysis (cf Supplementary Note 3). The time at which the fit translocation time distribution reaches its maximum is defined as the translocation time t, given as inset.

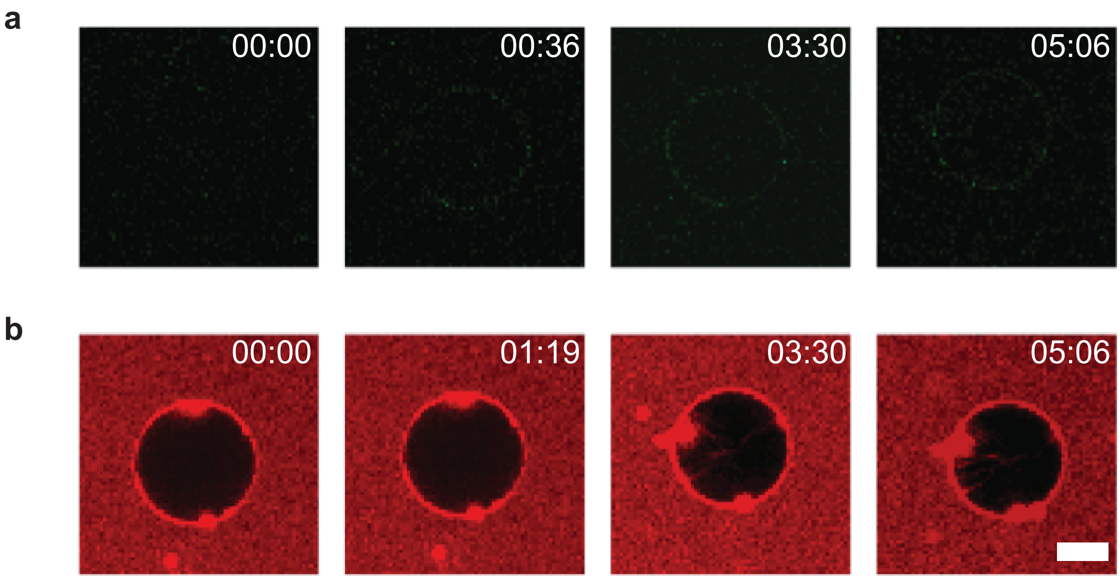

Supplementary Figure 17. Dye influx assay using the pin pore. a, Time series observing binding of fluorescently labelled pin pores to the vesicle. The increase of fluorescence over time at the boundary of the vesicle indicates continuous binding of pores to the vesicle. b, Time series monitoring Atto 633 dye influx into the vesicle after external addition of the pin pore. The lack of dye influx indicates that the pin pore is not penetrating the membrane. Note growth of filaments on the vesicle over time indicating membrane destabilisation or deformation upon binding of pores. Dye influx is not observed. Scale bar: 5 µm.

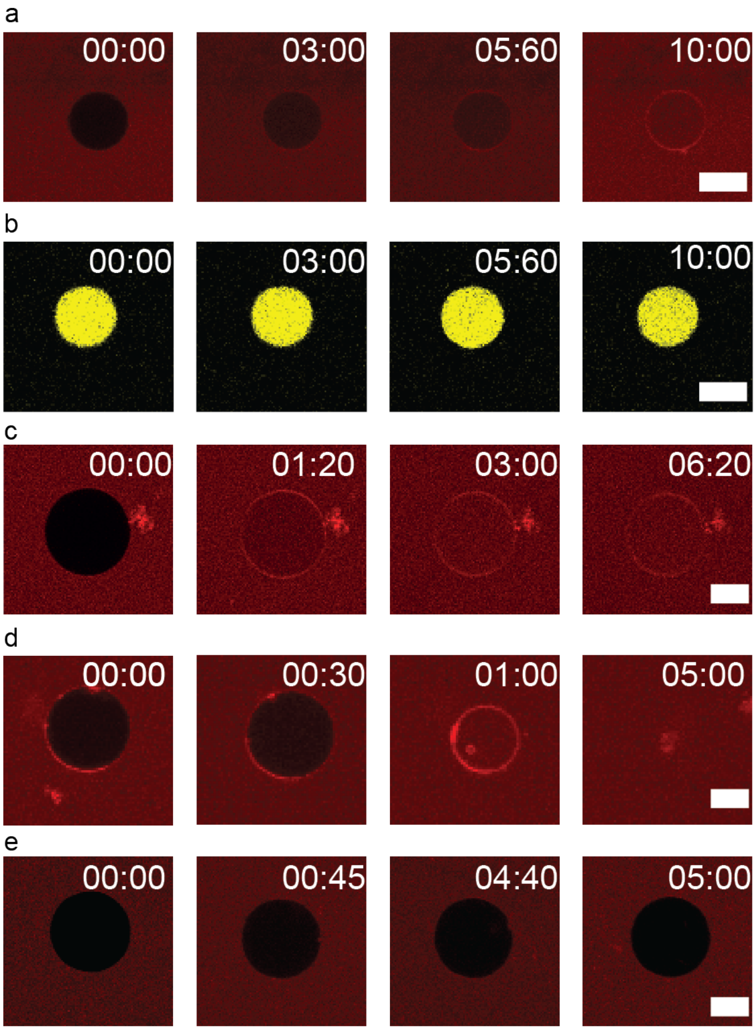

Supplementary Figure 18. Dye influx assay using the wheel pore. a, Time series of Atto 633 dye influx into a vesicle containing encapsulated wheel pores. b, Time series of the same vesicle, in which the fluorescence of a dextran-fluorescein conjugate was observed. The bulky dextran conjugate (with a nominal radius of 14.7 nm) is unable to enter the vesicle through the pore. c, Time series of dye influx induced by externally added wheel pores. d, Example for a time series with externally added wheel pores resulting in membrane destabilization. In this time series, the volume of the vesicle changes post addition of the pores followed by a bursting of the vesicle. e, Control experiment. In the absence of DNA origami pores the membrane is not permeable to the dye. Scale bar: 10 µm.

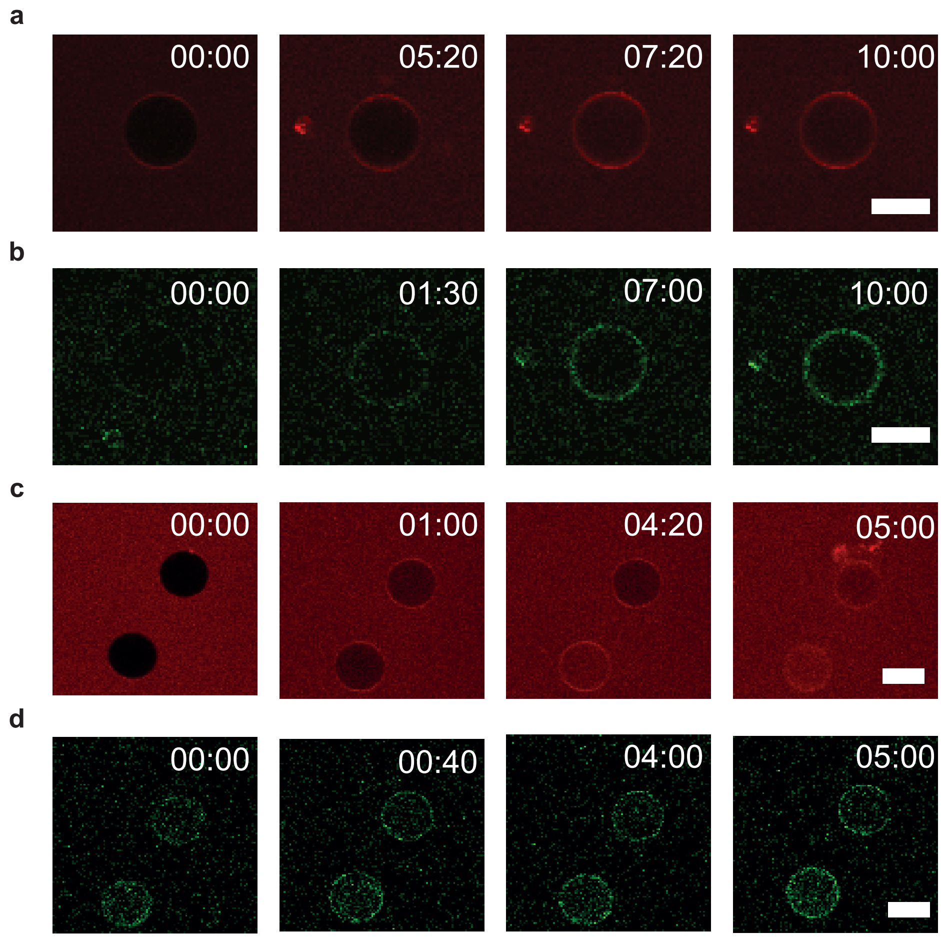

Supplementary Figure 19. Dye influx assay with the T pore. a, Time series of dye influx into a vesicle indicating membrane perforation by externally added pores. b, Time series showing fluorescently labelled origami pores binding to the same vesicle as in a,. c, Time series of dye influx observed for vesicles with encapsulated pores. d, Time series showing fluorescently labelled origami pores encapsulated within the same vesicles as in c,. Note that fluorescently labelled pores are present at the vesicle boundary from t = 0, in contrast to the externally added T pores in b, Scale bar: 10μm

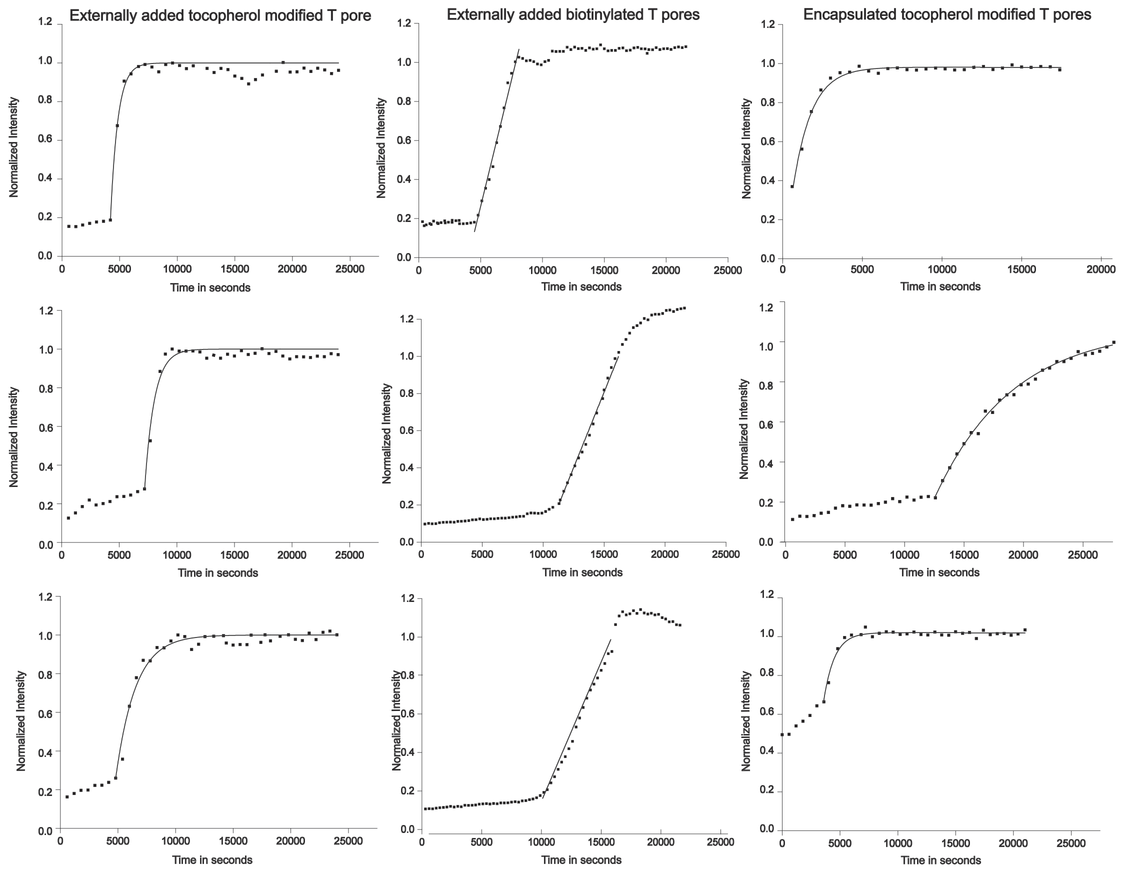

Supplementary Figure 20. The first two columns show example traces demonstrating the increase of fluorescence inside the vesicles when T pores modified with tocopherol or biotin are added externally. The third column shows traces for tocopherol modified pores, which were encapsulated. The traces are fit using an exponential model or its first order approximation.

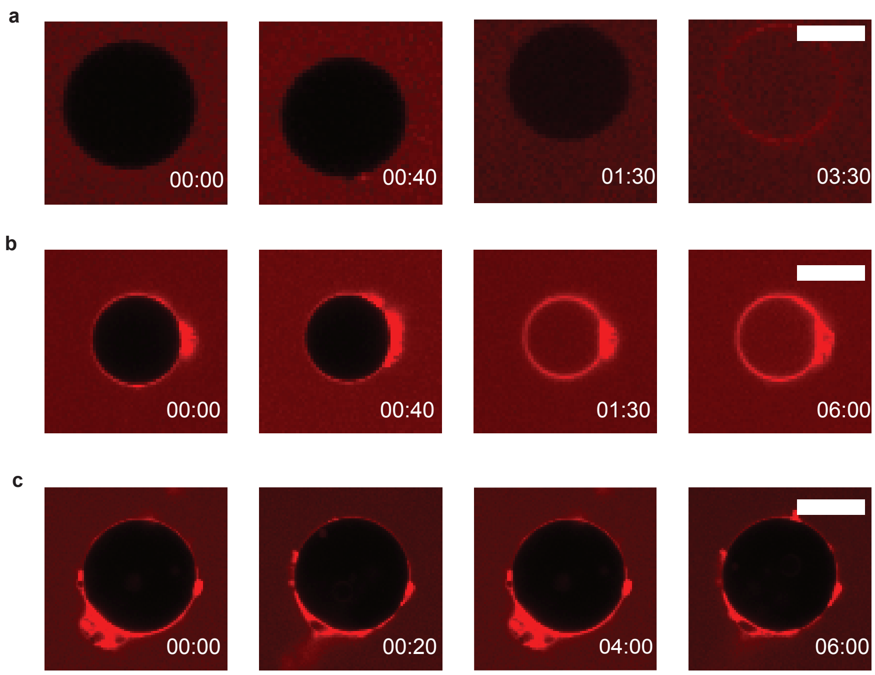

Supplementary Figure 21. Dye influx assay using the naturally occurring protein pore alpha hemolysin a, Time series of monitoring dye influx inside the vesicle using alpha hemolysin at a final concentration of 0.59 μM (subunits) or 84nM of full pores ; b, Time series of monitoring dye influx inside the vesicle using the alpha hemolysin pore at a final concentration of 3.78 μM of subunits or 540 nM of full pores c, Control vesicle without addition of pores observed over the time of the experiment. Many vesicles burst at these concentrations, due to high incorporation frequency, which can cause membrane deformation. TEM images confirm high level of interaction between the pores and SUVs. Concentration differences can be seen in the graph following dye influx during the time of the experiment. Scale bar: 50 nm.

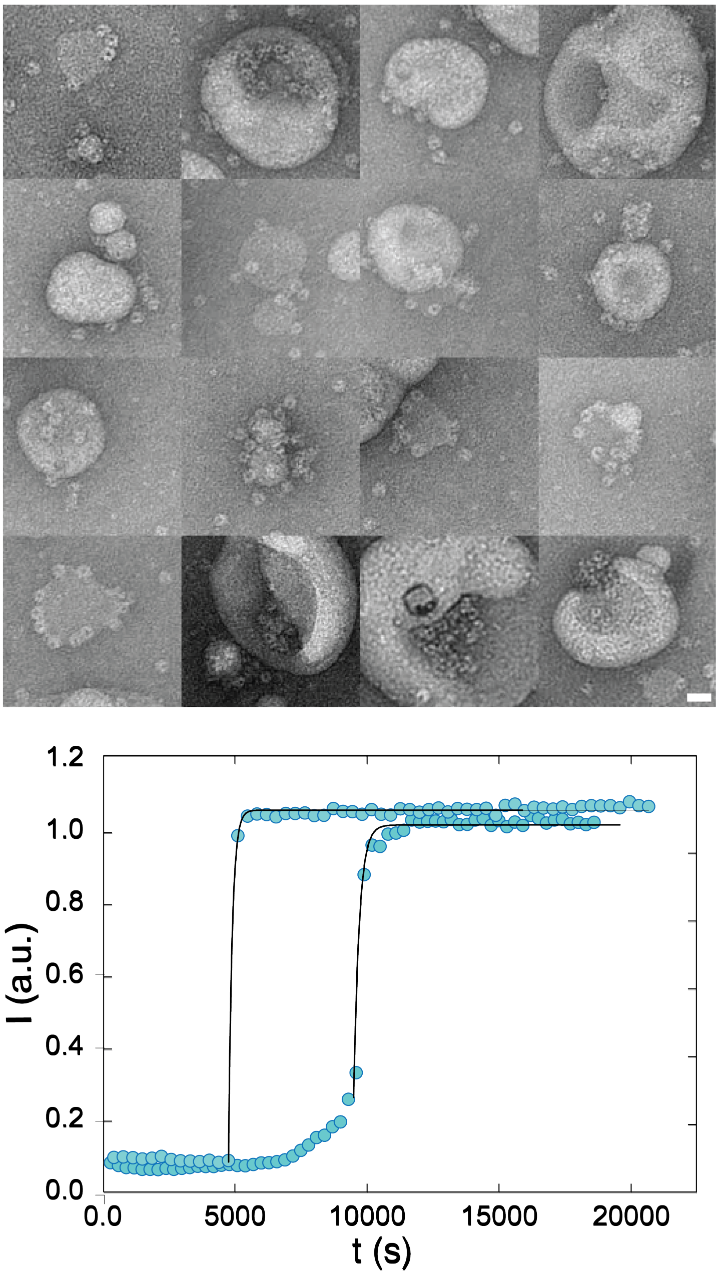

Supplementary Figure 22. a, TEM images of alpha hemolysin interacting with SUVs. Note the large number of pores localized on the vesicles. Scale bar: 50 nm. b, The graph shows dye influx into the vesicle after addition of alpha hemolysin at (monomer) concentrations of 3.74 μM and 0.59 μM. The graph shows no influx first, followed by a sudden jump in fluorescence. Lower concentration of subunits results in a longer waiting time.

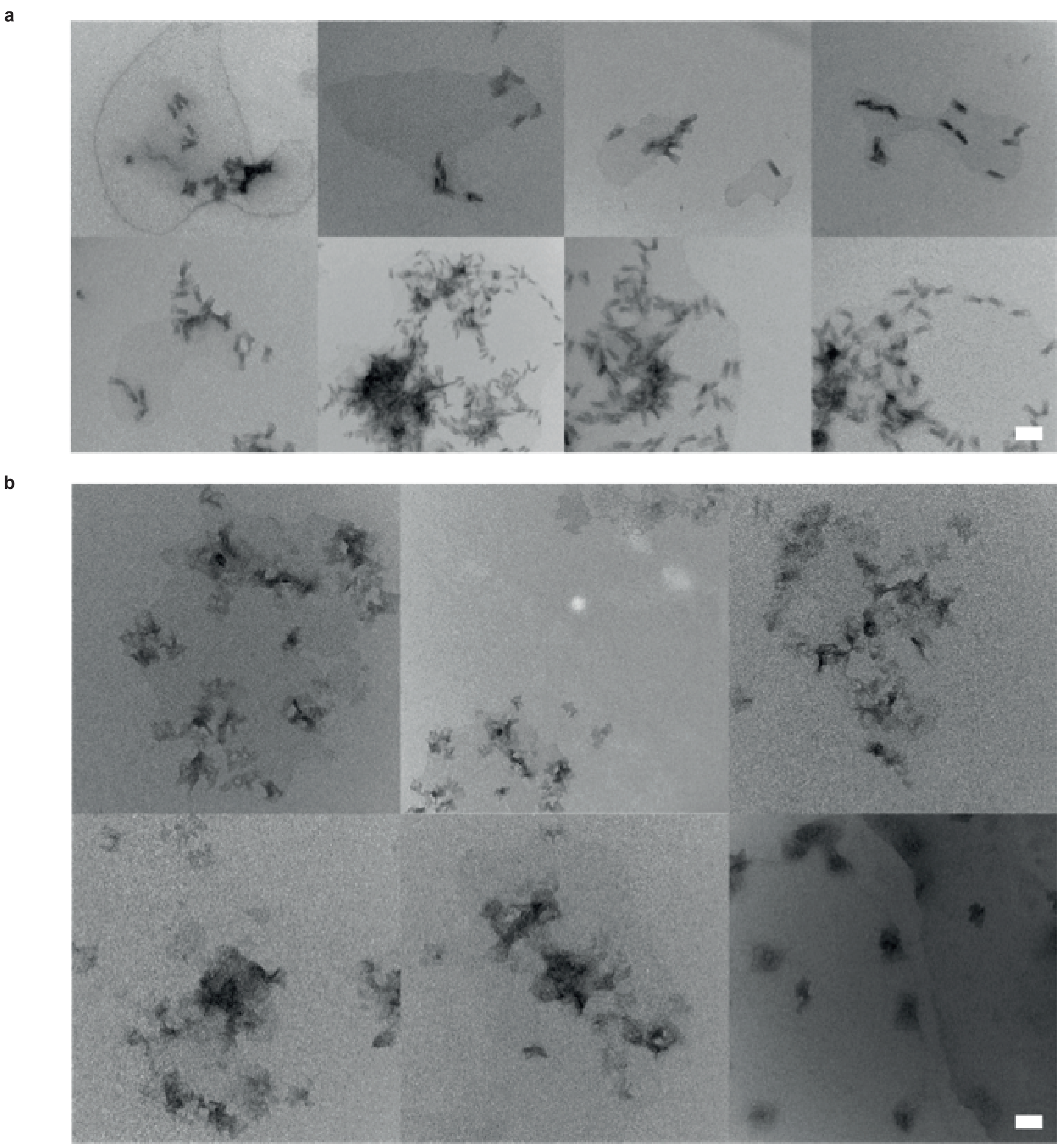

Supplementary Figure 23. a, TEM images of pin pores encapsulated in GUVs b, TEM images of T pores encapsulated in GUVs. Scale bar: 50 nm

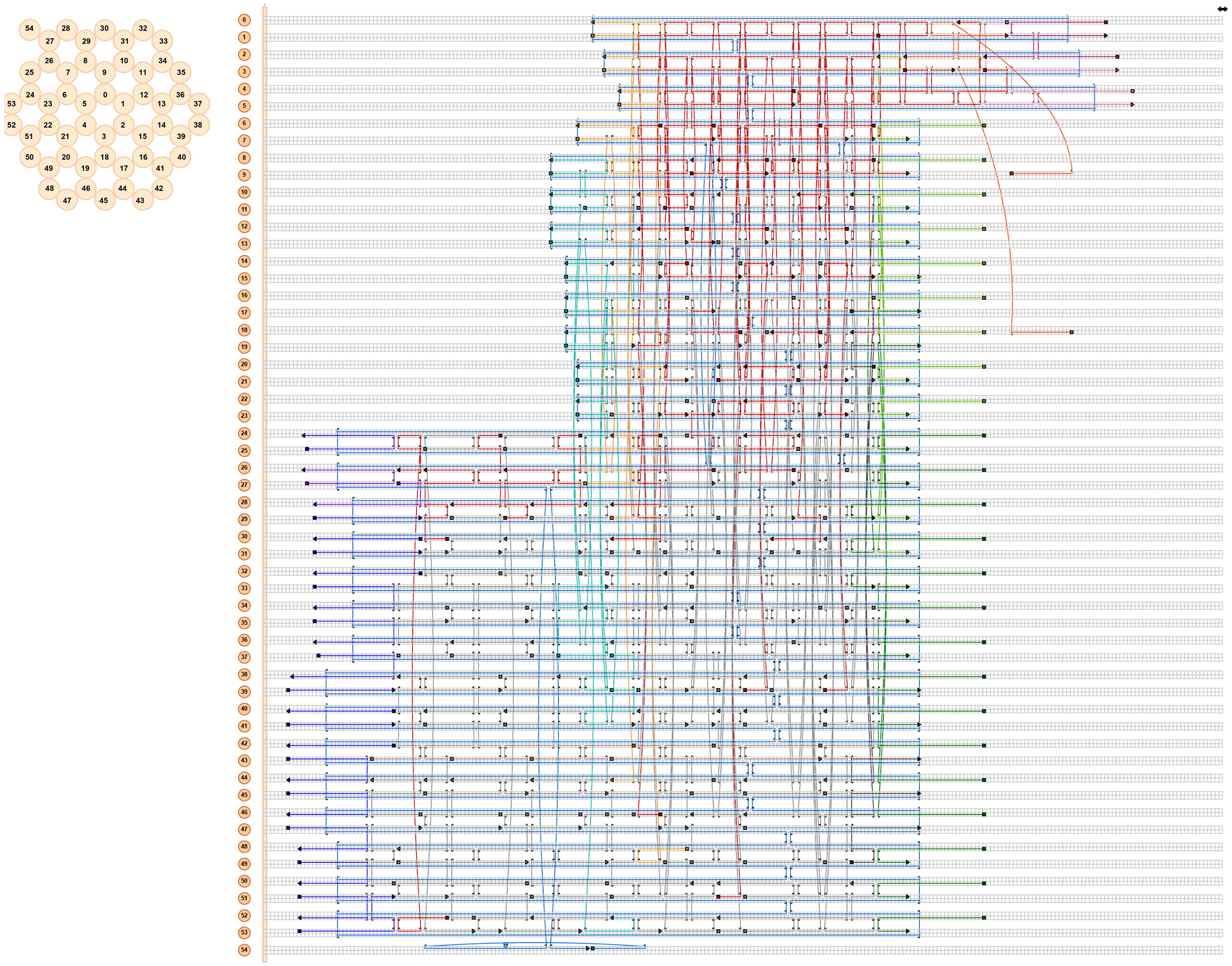

Supplementary Figure 24a: caDNAno design for the pin pore.

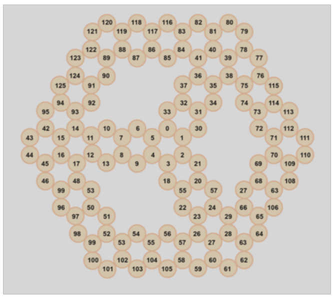

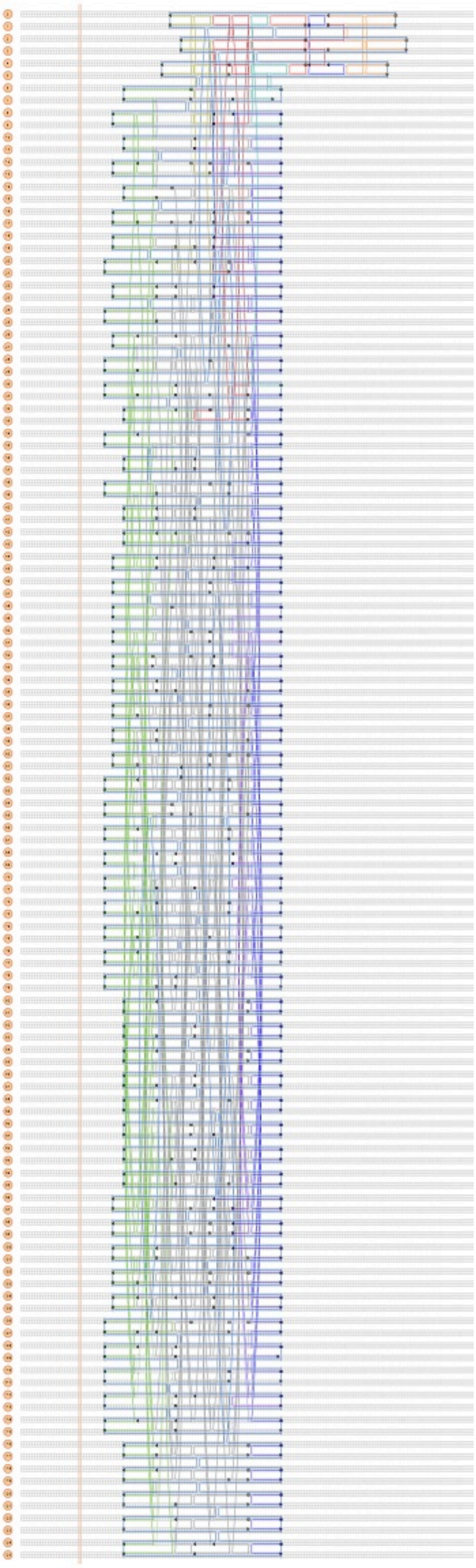

Supplementary Figure 24b: caDNAno design for the wheel pore

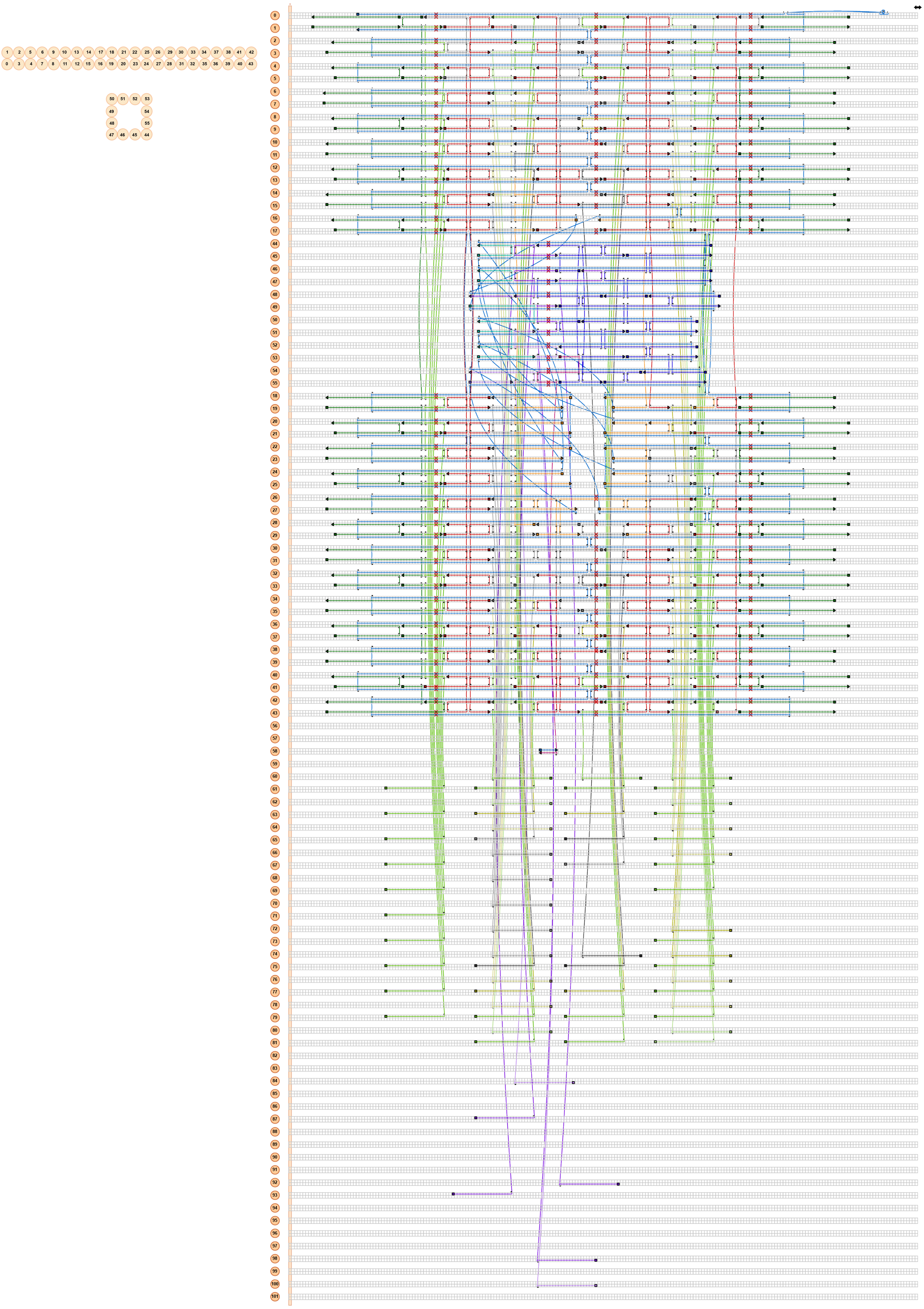

Supplementary Figure 24c: caDNAno design for the T pore.

	pin	wheel	T-pore
Stem length l (nm)	42	28	32
Cross-sectional area, theoretical (nm^2)	4.5	4.5	15.5
Conductance, experimental (nS)	1.6	1.5	3.1 ± 0.3
Cross-sectional area, experimental (nm^2)	4.4	4.2	10.9 ± 1.1

Supplementary Table 1: Geometrical dimensions and conductance of the pin, the wheel and the T pore

Supplementary Note 1: Design and fabrication of DNA origami pores

1) Rationale behind the pore designs:

One of the main goals of the present study is the creation of a series of DNA origami pores with improved, consistent, and stable membrane incorporation characteristics. Compared to our previous (first) generation of DNA membrane channels[1], two major changes were made. The new designs avoided unhybridized (single-stranded) scaffold loops, which potentially lead to unstable electrical properties and gating. Furthermore, we increased the number of anchor modifications in order to increase the incorporation frequency into the lipid bilayer (cf. Supplementary Figure 5). The caDNAno designs and the dimensions for all three pore variants are provided in Supplementary Figure 24 and Figure 1 respectively.

- The “pin pore” is based on the original DNA channel[1], but does not contain any unhybridized scaffold loops. In contrast, the previously unused part of the scaffold is rerouted to create a vestibule at the pore entrance similar as in α-hemolysin. The structure is folded using the 7249 nt long M13mp8 scaffold and is made of 54 double helices with 26 modification positions. The membrane spanning stem has a length of 14 nm and an inner diameter of 2.4 nm. The positions containing single-stranded handles for hybridization with adaptor sequences with tocopherol or biotin moieties are indicated in Supplementary Figure 5 a. TEM images of the folded pin pore are shown in Supplementary Figure 1, 6, and membrane interactions are demonstrated in Supplementary Figures 1 d , 7 and 8.
- The wheel pore is designed (like the pin pore using the honey comb lattice) to have a cap structure with a larger area for membrane interactions and a correspondingly larger number of anchor modifications (Supplementary Figure 5 b) and is based on a 8064 nt long DNA scaffold. As for the pin pore, single-stranded scaffold loops were avoided. The wheel structure has a large number of parallel helices (126), which enable a total of 57 modifications at the bottom of the cap. With 14 nm, the membrane spanning stem has the same length as for the pin pore, but the structure does not contain a vestibule. The holes between the axes of the wheel structure were designed in order to improve the folding properties of the membrane pore (there are no double helices deeply buried within the structure) (for TEM images see Supplementary Figures 1 g, h, 9 & 10)
- In contrast to the other pores, the T pore is based on a square lattice design and uses a 7560 nt long scaffold. The T pore consists of two functionally distinct parts. The top of the structure is a square-shaped plate with a side length of 51 nm. The top layer of the plate contains a rectangular hole with dimensions 3.7 nm x 10.4 nm = 38.6 nm^2 in the center, whereas the bottom layer has a hole with an area of A = 55.5 nm^2. The transmembrane stem of the pore is attached perpendicularly to the plate at the center of the bottom layer. The stem has a nominal length of 32 nm and a square inner diameter of ≈ 4.6 nm x 4.6 nm = 21.2 nm^2. At the bottom of the plate, 57 anchor modifications are introduced in order to facilitate binding of the structure to the lipid bilayer membrane (Supplementary Figure 5 c). The design is expected to facilitate particularly tight binding of the structure to the membrane – TEM images with SUVs even indicate bending of the plate along with the curvature of the vesicles (TEM images are shown in Supplementary Figure 1 k, 1 l, 3 & 4)

The DNA origami pores are synthesized in a one-pot reaction from a reaction mix containing scaffold (50 nM) and DNA staple strands (150nM) in folding buffer (1 x TAE, pH 8.5, 5mM $MgCl_2$). The reaction mixtures were annealed in a TEDRAD (MJ Research, now Biorad) thermal cycling device starting with 65°C for 15 min, followed by a thermal ramp from 60° to 30°C, decreasing temperature 1°C per 60 min. After folding, the DNA nanostructures are

filter-purified with 100 kDa Amicon ultra filters (Millipore) to filter out the excess staples in several rounds of buffer exchange (500 mM KCl to 1M KCl, 1 x TAE, pH 8.5, 5mM $MgCl_2$). The origami structures were stored at room temperature.

For anchoring the DNA nanopores into lipid membranes, the pores are designed to have DNA handles (single-stranded staple extensions) at specific positions, which are sequence-complementary to appropriately functionalized adaptor molecules. Tocopherol-modified DNA origami channels are thus prepared by incubating unpurified DNA nanostructures with an excess of adaptor oligonucleotides functionalized with a tocopherol moiety at their proximal end (5') for 45 minutes. After hybridization with the adaptor sequence, unbound staples are removed by filter purification as described.

In order to produce biotin-streptavidin modified DNA channels, the adaptor sequence is replaced with a sequence containing a biotin molecule instead of a tocopherol. The structures are purified to remove excess biotinylated staples using 100 kDa filters. Streptavidin is added to the structures without eluting in the same filter at a concentration of 10 µM for 45 minutes. A ten-fold excess of the streptavidin molecules is maintained to avoid binding of a single streptavidin to several neighbouring biotin molecules. Further rounds of filter purification remove excess streptavidin and staples. In TEM images, the overall shape of the structure is maintained (Supplementary Figure 3b, 4b, 6b, 8, 9b, 10b).

Supplementary Note 2: Electrical characterization

1) Conductance values

The conductance expected for a simple pore with length l, diameter d, and cross-sectional area A equals

$$G = \sigma \left(\frac{l}{A} + \frac{1}{d}\right)^{-1} \quad (1)$$

Here the first term in the bracket corresponds to the contribution of the conductive channel itself, while the second term accounts for the access resistance. For a 1 M KCl electrolyte the conductivity σ is 9.9 S/m [27].

The cross-sectional area of the pore determines the conductance value. The area for the pin and the wheel pore A_1 is shown in Supplementary Figure 2a. The theoretical cross-sectional are for the T pore is shown in Supplementary Figure 2b. The stem area is masked slightly by the top layer, therefore the *lowest* cross-sectional area A_2 through which the ionic current has to pass equals the intersection of stem area and the hole in the top layer.

The geometrical dimensions of the pore as well as the experimentally determined conductance of each pore are displayed in Supplementary Table 1. To compare the theoretically expected area with the one, which can be reversely calculated from the experimental conductance, the experimentally extracted cross-sectional areas also are stated in Supplementary Table 1. The difference of theoretically expected and experimentally extracted cross-sectional area for the T-pore could be attributed to lipid molecules pressing

helices 46, 47, 51 and 52 ≈ 0.6 nm inside the stem region ($2 * 0.63\ \mathrm{nm} * 3.7\ \mathrm{nm} = 4.7\ \mathrm{nm}^2$). The helix numbers are the same as in Supplementary Figure 24c.

2) Conductance measurements

The conductance values of the pores were determined from I-V curves, which were recorded with a droplet-interface-bilayer (DIB) setup as described in Methods. The pin showed a very low incorporation probability, and thus only one I-V curve was obtained (Supplementary Figure 11a), whereas a much larger number of measurements was performed for the wheel pore (Supplementary Figure 11b, N=9) and the T pore (Supplementary Figure 11c, N=45).

For the wheel we obtained values around 1.5, 2.8 and 5.8 nS. With TEM it was observed that the wheel tends to aggregate in groups of two, three, four or more pores. We therefore assume, that the conductance values around 1.5 nS correspond to one pore, the values around 2.8 nS correspond to two pores and the values around 5.8 nS correspond to four pores.

The T pore mainly shows single incorporations. An example of a multiple pore incorporation is shown in Supplementary Figure 12. Here the total conductance of all incorporated pores equals 27 nS. A voltage ramp with 1 V/s starting at +100 mV was applied, while the two droplets were separated from each other. By separating the droplets, the bilayer is split back into two monolayers. During this process, the conductance reduced in a stepwise fashion, showing nine distinguishable steps, indicating that the pores sequentially dissociate from the lipid bilayer. The conductance values of the pores can be derived from each of the steps.

The conductance was not influenced by the type of anchoring, as shown in Supplementary Figure 13. For electrical measurements with biotin-streptavidin anchoring 2% of the DPhPC was replaced by biotinylated DOPE.

Supplementary Note 3: DNA translocation experiments

1) Translocation of DNA through the T-pore - theory

The translocation times and velocities were extracted by regression analysis of the dwell-time histograms based on a 1D drift diffusion model[2], which has been previously applied to nanopore translocation experiments[4, 5, 6, 7, 8, 9, 10, 11]. According to the model the probability $P(t)$ that a translocation event has length t is given by:

$$P\ (t) = \frac{b}{(4\,\pi\, Dt^3)^{1/2}} \times \exp\left(-\frac{(b - v\ t)^2}{4D\ t}\right) \qquad (2)$$

Here, the parameter b is the sum of the length of the pore and the contour length of the translocating DNA molecules, D is the diffusion coefficient for 1D diffusion of DNA through the pore (or the pore along the molecule) and v is the drift velocity. Diffusion coefficient D and velocity v are obtained from regression analysis of our data. The time for which this translocation time distribution reaches its maximum (the mode) is defined as the translocation time.

2) Experimental determination of translocation events - from recorded traces to event detection

All current traces are recorded with a 200 kHz bandwidth corresponding to a temporal resolution of 5 µs per data point. Current traces are median-filtered to further reduce noise. Each point of a filtered trace equals the median over the 20 previous points of the original trace, hence the maximum resolution is reduced to values higher than 50 µs. The median filter affects the heights of the peaks, but conserves the durations of the events, which is the more important parameter for our dwell time analysis, and which is used to prove DNA translocation through the pores. Real translocation events are characterized by an inverse proportionality between event length and applied voltage ($\tau \sim 1/\mathrm{V}$), which is not observed for gating or other current fluctuations.

The detection of translocation events is performed using a custom-written Matlab routine. The average current over 1000 data points (= 5 ms), 700 points ahead of the event is taken as an upper baseline. Every point that deviates from this value more than a preset threshold*10 is counted as the beginning of an event. The end of the event is defined, when the current returns to the upper baseline within the preset threshold*2.

The manually chosen threshold increases with voltage. As measurements of the background signal indicate a roughly linear increase of noise with increasing voltage, the threshold is also increased linearly with voltage. The difference between upper baseline and lower baseline defines the depth of the event *ΔI*.

Translocation experiments with PEG rendered the droplets viscous, translocation experiments were performed with dsDNA.

3) Translocation of dsDNA (527 bp) through the T pore

The set of translocation experiments shown in Figure 2 of the main paper was performed with 527 bp long dsDNA analytes (sequence 5' ATACCATGGATATGGGCGGCCACGGCGTGGGTGTAC CAGGAGTAGGAGTACCAGGAGTAGGAGTACCAGGAGTAGGAGTACCAGGAGTAGGAGTAGCAGGAGTGCCGGGCG

TGGGTGTACCAGGAGTAGGAGTACCAGGAGTAGGAGTACCAGGAGTAGGAGTACCAGGAGTAGGAGTAGCAGGAG TGCCGGGCGTGGGTGTACCAGGAGTAGGAGTACCAGGAGTAGGAGTACCAGGAGTAGGAGTACCAGGAGTAGGAG TAGCAGGAGTGCCGGGCGTGGGTGTACCAGGAGTAGGAGTACCAGGAGTAGGAGTACCAGGAGTAGGAGTACCAG GAGTAGGAGTAGCAGGAGTGCCGGGCGTGGGTGTACCAGGAGTAGGAGTACCAGGAGTAGGAGTACCAGGAGTAG GAGTACCAGGAGTAGGAGTAGCAGGAGTGCCGGGCGTGGGTGTACCAGGAGTAGGAGTACCAGGAGTAGGAGTAC CAGGAGTAGGAGTACCAGGAGTAGGAGTAGCAGGAGTGCCG 3‘). In Fig. 2c, a scatter plot of the current reduction ΔI vs. the translocation time τ is shown for all events detected at 75, 100 and 125 mV. Supplementary Figure 14 also shows the respective events at 75, 100 and 125 mV before the addition of the dsDNA.

The current reduction ΔI hands us a mean to draw conclusions about the area of the hole in the T-pore. Current reductions are normally distributed around a voltage specific ΔI (here 74 pA for 75 mV, 91 pA for 100mV and 116 pA for 125 mV), with relative current reductions $\Delta I/I$ of around 30% (Supplementary Figure 15). The current reduction is the consequence of the cross-sectional area being reduced to 70%, as 30% of the area are occupied by the dsDNA. Following the rule of proportion the cross-sectional area should approximately equal 11.5 nm^2.

The area of ≈ 11.5 nm^2 for the cross-sectional area of the T pore derived by $\Delta I/I$ is in accordance with the 10.9 ± 1.1 nm^2 extracted from the conductance of the T-pore (Supplementary Table 1).

4) Translocation of ssDNA (140 nt) and dsDNA (115 bp) through a modified T pore

Another set of translocation experiments was performed with both ssDNA and dsDNA analytes, using a T pore modified with a 6 nt long single-stranded DNA obstacle (sequence 5’ TTGGCC 3’) in the middle of the central channel. In the experiments, first ssDNA (5’-$(T)_{68}$CCGG$(T)_{68}$-3’) and then dsDNA (5’-ATAATACGACTCACTATAGGGCGCCGCGAAAA CGCGGCGCCCAAAAAAAAAAAAAAAAAAAAAAAAAAAAAAGGATCCCGACTGGCGAGAGCCAGGTAAC GAATGGATCCAAAAA-3’) molecules were added to one of the aqueous droplets of the DIB setup (2M KCl, 5mM $MgCl_2$) in a stepwise fashion.

While in the presence of only ssDNA translocation events were indeed recorded (Supplementary Figure 16d), their depth and length overlapped with events occurring in the background signal. However, the event frequency increased significantly compared to background. As expected for real translocation events, event frequency and translocation velocity increased (Supplementary Figure 16b), while translocation time decreased with increasing voltage (Supplementary Figure 16e). The diffusion coefficients (2.4 nm^2/µs (50mV), 2.4 nm^2/µs (75 mV) and 2.8 nm^2/µs (100 mV)) were approximately independent of the applied voltage.

Clear translocation events were observed for the ssDNA-dsDNA mixture, i.e., after addition of dsDNA (Supplementary Figure 16g). The event frequency increased drastically compared to the previously recorded ssDNA (Supplementary Figure 16c). The translocation times decreased with increasing voltage (from 184 µs (50mV) over 170 µs (75mV) to 150 µs (100mV)), whereas the velocity increased with voltage (0.26 nm/µs (50mV), 0.32 nm/µs (75mV) and 0.42 nm/µs (100mV)). These results confirm that the observed events are caused by the passage of DNA through the pore.

In order to obtain information about translocation time and velocity of the translocating dsDNA molecules only, the events were split into two populations for further analysis. We attributed the top (large ΔI) population in the scatter plots predominantly to the presence of dsDNA (Supplementary Figure 16i, top), while the bottom population (shown in faded colours in Supplementary Figure 16i) was not taken into account. The resulting translocation times obtained from the restricted population are displayed in Supplementary Figure 16h (velocities in Supplementary Figure 16c). The diffusion coefficients 2.7 $nm^2/\mu s$ (50mV), 2.5 $nm^2/\mu s$ (75 mV) and 2.3 $nm^2/\mu s$ (100 mV) obtained by the 1D drift-diffusion regression analysis (cf. Supplementary Note above), are approximately independent of voltage.

The velocity of dsDNA is thus approximately half of the values obtained for ssDNA in this particular setup. This finding seems consistent with the smaller persistence length, diameter and charge density of ssDNA, which might result in less friction and electrostatic interactions inside of the DNA channel.

Notably, the translocation of 115 bp dsDNA in the presence of an obstacle inside of the channel is much slower (by a factor of ≈ 5) than that of the longer 527 bp dsDNA without obstacle (cf. Figure 2d and Supplementary Figure 16c), indicating that the ssDNA obstacle has a drastic effect on translocation dynamics.

Supplementary Note 4: Dye influx assay

1) Experimental aspects

The dye influx assay is regularly used to study interactions of membrane binding peptides and pores with lipid membranes, and has here been adapted to characterize membrane perforation by DNA origami pores. The assay involves the long term observation of indvidual giant unilamellar vesicles (GUVs). Influx of an otherwise lipid membrane impermeable dye molecule into the vesicle indicates the formation of a membrane channel by the DNA origami structures. In order to be able to perform the studies, buffer conditions were carefully adjusted to avoid accidental bursting or perforation of the GUVs.

Stable giant unilamellar vesicles are predominantly made using the inverted emulsion technique[8] or also the related cDICE (continuous droplet interface crossing encapsulation) method. The advantage of these techniques is that a wide range of solutions can be encapsulated inside the vesicle provided the external solution has the same osmolarity. By contrast, electroformation of GUVs has limitations on the salt conditions used for the internal solutions and cannot be used for encapsulation of DNA origami structures inside of vesicles.

The GUVs were produced as described in Materials and Methods in the main paper. Lipids used to form the initial uniform film were Egg PC (L-α-lysophosphatidylcholine) (90%) and biotinylated DOPE (1,2-dioleoyl-sn-glycero-3-phosphoethanolamine-N-(biotinyl)) (10%). The internal solution consisted of sucrose solution (400mM) and KCl (500mM), while the external solution was composed of glucose (400mM) and KCl (500mM). The compositions of the solutions are chosen in such a way that the vesicles are heavier than their surroundings, which assists in sinking of the GUVs in the ibidi observation chambers. This ensures easier observation of the vesicles using the confocal microscope without compromising the stability of the vesicles. High monovalent salt concentrations were used to maintain the stability of the DNA origami structures, but divalent ions were avoided as they strongly reduced the stability

of the GUVs. The external addition of tocopherol-modified adaptors did not lead to leakage or instability in the GUVs (data not shown) probably due to larger size of the dye than the leak pathways in the bilayer produced by tilting of the lipid heads due to insertion of a single stranded DNA or a DNA duplex[12].

Encapsulation of the DNA origami structures was carried out by replacing the internal solution with 20 nM (final concentration) of origami structures along with sucrose and KCl. Immobilisation of the GUVs when using tocopherol modified structures is done by tethering biotinylated head groups to the bottom of biotin-streptavidin modified chambers. This strategy is not used when using biotin-streptavidin bridges for membrane incorporation in order to ensure that all the biotinylated head groups in the GUV are engaged in interactions with the streptavidin modified pore. The vesicles are then simply allowed to sink and incubated for one hour before imaging.

2) Dye influx kinetics

In order to quantitate the kinetics of dye influx, we assume that the flux j through a single channel is given by

$$j = D\frac{n_o - n_i}{L} \quad (3)$$

where D is the diffusion coefficient of the dye, L is the effective length of the channel, and n_o, n_i are the dye concentrations outside and inside of the liposome (in units of $1/\mathrm{m}^3$).

Thus the number of dye molecules N_i inside of the liposome increases as:

$$\dot{N}_i = j \cdot A = \frac{DA}{L}(n_o - n_i), \quad (4)$$

where A is the channel cross-section, and therefore the concentration changes according to:

$$\dot{n}_i = \frac{DA}{LV}(n_o - n_i) =: k_0(n_o - n_i) \quad (5)$$

where V is the volume of the vesicle, and we introduced the abbreviation $k_0 := DA/LV$, which has units of 1/s.

Now, assuming that the dye concentration in the solution surrounding the vesicle stays constant, we can solve for the concentration inside:

$$n_i(t) = n_o - \left(n_o - n_i(0)\right) \times e^{-k_0 t} \quad (6)$$

If the initial concentration inside is zero, $n_i(0) = 0$, this simplifies to:

$$n_i(t) = n_o(1 - e^{-k_0 t}) \quad (7)$$

This is the increase in dye concentration due to flux through a single channel.

Experimentally obtained time traces typically followed this monoexponential behavior (cf. Supplementary Figure 20). Assuming a dye diffusion coefficient of $D = 1000\ \mu m^2/s$, we can estimate the typical time scale of dye influx for a single channel with diameter $A = 16.7\ \mathrm{nm}^2$, length $L = 32\ \mathrm{nm}$, and a vesicle volume of $V = 1000\ \mu\mathrm{m}^3$ to be $\tau_{single} = 1/k_0 \approx 2000\ \mathrm{s}$.

In most of the experiments, however, we observed considerably faster influx time scales. As the experiments with dextran-conjugated fluorescein molecules rule out the formation of larger holes in the membranes, we have to assume that dye influx typically occurs through multiple channels at a time. We thus heuristically determined a *normalized influx rate* from the ratio between the expected single channel influx rate and the actually observed rate or $k/k0$ which is also given in Fig. 3f & I.

Interestingly, this indicates that apparently multiple channels insert into the bilayer membrane at roughly the same time (alternative fits with sums of exponentials or a stretched exponential did not improve the results). Further corroborating this assumption, we observe that larger vesicles (with larger surface areas) typically have a faster dye influx corresponding to more channels inserting (cf. Fig. 3f & I). At least for the experiments with externally added channels this suggests a scenario, in which the membrane is initially covered with a large number of membrane channels – potentially clustered or aggregated –, of which some simultaneously insert into the membrane in a concerted manner.

Simple influx kinetics is expected to be exponential, but not all traces observed could be well fit. For instance, images could only be taken every 5 or 10 minutes, and thus very fast kinetics was not very well resolved. In such cases, and also in cases with distorted traces, a linearized version of the exponential influx model was used to determine *k.* Addition of the origami pores externally modified with tocopherol or mediating membrane interaction via biotin streptavidin bridges displayed different kinetics in the dye influx assays. The biotinylated pores typically resulted in a slower influx of the dye, indicating a smaller number of pores interacting with the vesicle. The influx rate was determined using a linearized approximation of the dye influx model. Encapsulated tocopherol-modified pores more often showed an increased fluorescence at t=0, which indicates immediate influx into the membrane after encapsulation of the pores.

3) Dye influx assay with the pin pore

In dye influx experiments with the pin pore filling of the vesicles with the external dye was not observed (Supplementary Figure 17). In contrast to conductance measurements (where membrane incorporation is assisted by an electrical field) or experiments with SUVs (with a higher curvature), the pin pore does not appear to be able to penetrate GUV bilayer membranes spontaneously. Images recorded with fluorescently labelled pin pores (Supplementary Figure 17a) demonstrate a complete coverage of the GUVs with the structures, most probably lying on the membrane with their long sides.

4) Dye influx assay with the wheel pore

Results of the dye influx assay with the wheel pore are summarized in Supplementary Figure 18. Both encapsulated as well as externally added pore experiments displayed successful dye influx.

5) Dye influx assay with the T pore

Most of the dye influx experiments in this study were performed with the T pore. Vesicles generally tended to burst after ≈ 8 hours, which makes interpretation difficult whether membrane destabilization was caused by pore binding or other factors.

6) Dye influx assay: comparison with α-hemolysin

For comparison with a conventional protein pore former, dye influx experiments were also performed using α-hemolysin (Sigma). In contrast to the DNA channels, the naturally occurring pore is a multimeric pore composed of 7 subunits and has a hydrophobic belt to interact with the lipid bilayer. TEM images of the aHL pore with SUVs show large numbers of pores interacting with the vesicle simultaneously (similar to tocopherol-modified DNA channels).

The dye influx itself showed a prolonged waiting time after addition of the channels, followed by rapid dye influx. Larger aHL concentrations resulted in shorter waiting times. While the waiting times are comparable to those observed for DNA origami channels for similar concentrations, the underlying membrane incorporation process is quite different, as the formation of the α-hemolysin pore also involves multimerization and pre-pore formation.

Supplementary Note 5: Encapsulation of origami pores into GUVs.

The encapsulation of origami pores and dextran dye into GUVs is carried out using the inverted emulsion technique (Materials and Methods). The internal solution is replaced to produce the required solution containing 500 mM KCl, 400 mM sucrose and either 6 µM dextran dye or 20 nM origami pores. The external solution is modified to have similar osmolarity and a final glucose concentration of 400 mM to allow the GUVs to sink. The GUVs solution is used directly on the TEM grids and imaged. The pin pore is easy to observe under the burst GUV (Supplementary Figure 23a) and the T pore can be spotted as well (Supplementary Figure 23b). This along with the presence of fluorescent vesicles when encapsulating the dextran dye conjugate indicates successful compartmentalization of required molecules into GUVs while maintaining their functionality.

Supplementary References

1. Langecker M, *et al.* Synthetic lipid membrane channels formed by designed DNA nanostructures. *Science* **338**, 932-936 (2012).

2. Hall AR, Scott A, Rotem D, Mehta KK, Bayley H, Dekker C. Hybrid pore formation by directed insertion of [alpha]-haemolysin into solid-state nanopores. *Nat nanotechnol* **5**, 874-877 (2010).

3. Larkin J, Henley RY, Muthukumar M, Rosenstein JK, Wanunu M. High-bandwidth protein analysis using solid-state nanopores. *Biophys J* **106**, 696-704 (2014).

4. Carson S, Wilson J, Aksimentiev A, Wanunu M. Smooth DNA transport through a narrowed pore geometry. *Biophysical journal* **107**, 2381-2393 (2014).

5. Carson S, Wick ST, Carr PA, Wanunu M, Aguilar CA. Direct analysis of gene synthesis reactions using solid-state nanopores. *ACS nano* **9**, 12417-12424 (2015).

6. Carson S, Wilson J, Aksimentiev A, Weigele PR, Wanunu M. Hydroxymethyluracil modifications enhance the flexibility and hydrophilicity of double-stranded DNA. *Nucleic acids research*, gkv1199 (2015).

7. Zhou Z, *et al.* DNA translocation through hydrophilic nanopore in hexagonal boron nitride. *Sci Rep* **3**, 3287 (2013).

8. Pitchford WH, *et al.* Synchronized optical and electronic detection of biomolecules using a low noise nanopore platform. *ACS nano* **9**, 1740-1748 (2015).

9. Wu H, *et al.* The Estimation of Field-Dependent Conductance Change of Nanopore by Field-Induced Charge in the Translocations of AuNPs-DNA Conjugates. *The Journal of Physical Chemistry C* **118**, 26825-26835 (2014).

10. Sze JY, Kumar S, Ivanov AP, Oh SH, Edel JB. Fine tuning of nanopipettes using atomic layer deposition for single molecule sensing. *Analyst* **140**, 4828-4834 (2015).

11. Angeli E, *et al.* Simultaneous Electro-Optical Tracking for Nanoparticle Recognition and Counting. *Nano Lett* **15**, 5696-5701 (2015).

12. Göpfrich K, *et al.* Ion Channels Made from a Single Membrane-Spanning DNA Duplex. *Nano Letters* **16**, 4665-4669 (2016).
