# Supplementary material for: Molecular transport through large-diameter DNA nanopores
Source: Nat Commun. 2016 Sep 23;7:12787. doi: 10.1038/ncomms12787 (PMC5036142; doi:10.1038/ncomms12787)
Supplement: Supplementary Dataset 1 — DNA staple sequences for the origami membrane channels [file ncomms12787-s2.docx]

| **Pin pore** |  | |
| --- | --- | --- |
| Start | End | Sequence |
| 0[195] | 3[224] | TCAGGCTGGTTGATAATCAGAAATATTTGTGAGCGAGTAACAACCCGTTTTTTTTTT |
| 0[221] | 2[189] | TTTTTTTTTTAAGCGCCATTCGCCATTGCCGGATGGGAACAAACGGC |
| 1[161] | 3[181] | TCGCACTCCAGCCAAAGGGCGCATGTCAATCATATAATATTTCAGCTTT |
| 1[86] | 2[89] | TTTTCCCAGTCACTAGAGGAT |
| 10[189] | 31[169] | TAACAGGATTAGCAGAGCGAGGTTCATCAAGAGTAATAGGAAC |
| 11[105] | 7[118] | AGCGAGATTACGAGTGCAGATACGGAACCGGGAAACCTGTAT |
| 11[112] | 34[112] | GGCTTTTGCAAAAAAGATATTTTTGAAT |
| 11[154] | 40[147] | GCGCATACGGTCAATCCATGTCGCGAAACCAGTAACTCATTAAAGCCAG |
| 11[75] | 33[90] | CGTTTACCATAGCCCTGATAGAACCCTTCT |
| 11[84] | 8[75] | GACGACGACACTATAGGAATATAGAAAGATTCATCAG |
| 11[98] | 32[105] | CCAAAATAGTCTTTAAGCGTAAGAATACATATTAC |
| 12[111] | 23[111] | AGAGGGGGTAAAACGGCGATTACGAGCCATGAGTGCCAATAA |
| 12[132] | 5[139] | CAACTTTTTGAGGGCGAAAGGTAAGCAAACAAGAG |
| 12[153] | 23[153] | TCATAAGCGGCCTCCTTCGCTGAACGGTTATGCGAGAGCCTT |
| 12[189] | 11[169] | TAACAGGATTAGCAGAGCGAGGACGAGGCGCAGAGGCTGGCTG |
| 13[119] | 39[111] | AAATGTGAAACCCTCAATCAATTATTAAGGAACAAAGAAACCTCAGATG |
| 13[75] | 34[84] | TCCAATACTGAAAGGAGCAAATGAAAAATCGAACTGA |
| 14[104] | 34[105] | CGAGAATAATATTCTCAGTTGTCACCTTGCTGAACGGCTATT |
| 14[111] | 21[111] | CAGAAAAGAAGCAACCTGCAGCGAATTCATTGTTATGACCAT |
| 14[125] | 41[111] | CCCTTTAAACAGTTAATATACTAACGGATTCGCCTTAACCTT |
| 14[146] | 35[153] | CAACGGAAATCCGCAACCTATGACTCCTCAAGAGA |
| 14[189] | 13[169] | TAACAGGATTAGCAGAGCGAGGGATTATACCAAGTACTTAGCC |
| 15[79] | 41[97] | GGTCTTTACCCTGACTATTGACTTCATATATGT |
| 16[111] | 20[105] | GAGGAAGCCGGAAGTTTAATTAATATAATTTCATT |
| 16[139] | 40[126] | AAGGCACTAGGTTGAGGCAGGAATGGAAAGCGCACATCGGGA |
| 16[160] | 44[147] | AAAATACATTGGCCCAGAGCCGCCGCCAAGAGCCAGGAACCG |
| 16[189] | 15[172] | TAACAGGATTAGCAGAGCGAGGCATTAAACGGGTTTGACCCCCAGC |
| 17[154] | 43[172] | TTTTTCACACCACCCCACCCTCAGAGCCGCCAC |
| 17[79] | 18[79] | TAATTCGAGCTTGAGGTCATTTTT |
| 18[125] | 15[125] | GATTAGATGGTCATCACAAGCTTGCATGAGCGGATTGCATAC |
| 18[132] | 46[126] | AACGGAGATGTTTTAAATATTCATTTTCGGTCAAG |
| 18[146] | 11[146] | GGAACGACATCAAAGCATCGTACAGTATGGAACCGGGTGTAC |
| 18[189] | 2[161] | TAACAGGATTAGCAGAGCGAGGCACCCTCAGCAGGGCCTTCGTCACGT |
| 18[212] | 1[195] | TAACAGGATTAGCAGAGCGAGGCATCAACGGATTGAGGCACCGCTTCTGG |
| 19[79] | 20[82] | GCGGATGGCTTAATTCCCAAT |
| 2[224] | 1[221] | TTTTTTTTTTTCGGATTCTCCGAACCAGGCATTTTTTTTTT |
| 20[160] | 45[172] | CCGATATGCCGCTTACTGTAGTTTTCATAATCAAAATCAC |
| 20[189] | 21[169] | TAACAGGATTAGCAGAGCGAGGGCCCACGCATAAACAACAACC |
| 21[119] | 9[132] | ATTTCGCGTTTCCTTAAAGTGGGGATGTGCTAACAAAGCTGC |
| 21[140] | 10[140] | CCGATAGAATCAGCAATCGATATTACGCGTGAATACCGGATA |
| 21[82] | 22[82] | TCTGCGAACATCAATTCT |
| 22[111] | 53[104] | GGCGCGAAGCCCAATTTAAGAAAAGTAAAGACACC |
| 22[118] | 46[112] | CATTTGGTAGATACCTGGAAGTGCTGTAAGTACCG |
| 22[153] | 24[140] | TTCTTAAAAATTATAGGGAGGGAAGGTAATTCATAAAAAAAA |

| 22[189] | 23[169] | TAACAGGATTAGCAGAGCGAGGGCTTGCTTTCGAATCGGTTTA |
| --- | --- | --- |
| 23[82] | 52[91] | ACTAATAGTCCAAAAATAAAAGAAACGCAAGCAGATA |
| 23[91] | 19[97] | AGTAGCAAGGTGGCGAGTAGAACAGTTGGAGCTTA |
| 24[104] | 28[91] | CTAAATCTCAAATCATTAATGTCAAGTTCCGATTTAGAGCTT |
| 24[111] | 50[112] | CATAAAGACGGAATAAGTTTAAGCCCTTTAATAAGTTATCCC |
| 24[153] | 50[154] | ATCTCCATGGTTTACCAGCGCCCGATTGTCATTAATTGAGCC |
| 24[189] | 25[169] | TAACAGGATTAGCAGAGCGAGGTAATTTTTTCACAATTGCGAA |
| 24[62] | 28[49] | TTGCGGGGTGTAGGTTGGGCGTGCAGCAAAAGGAAGGGAAGA |
| 24[83] | 28[70] | CATTATGGGTGAGACGGGGAGCTACGTGAAAGCCGGCGAACG |
| 25[11] | 24[10] | TTTTTTTTGAACCCTCATATATTTAGGATAAAAATTTTTATTTTTTTTT |
| 25[140] | 21[160] | AATTAATGCCGGAACTAAAGGGTTGAAATAATTGTGGTGAATGACAATG |
| 25[42] | 53[55] | AATGCCTGAGTAATAGAAGCCTTTATTTGTTAGCAAACGTAG |
| 25[63] | 52[70] | TAAAGATTCAAAAGACCCTGTAATACTTATACATAAAGGTGGCCAGAAG |
| 26[118] | 23[118] | CTCGTGCCAGCTGCACCATCAATATGATCTCAGAGATCATAC |
| 26[139] | 30[133] | TTTTTGAAGGCTCATTATACCTTGGGAAATTCCACCAGTACAAACTACA |
| 26[153] | 29[139] | ACAGAGGGTAGCTAGGATTTTCGTTAGTAAATGAACTGTAGC |
| 26[189] | 27[169] | TAACAGGATTAGCAGAGCGAGGGGAGTGAGAATAAACAGTTTC |
| 27[11] | 26[10] | TTTTTTTTCAGCTGATTGCCCTTCCCAGTGAGACGGGCAATTTTTTTTT |
| 27[35] | 28[13] | ACCGCCTAGGAGCGGGCGCTAGGGCGTTTTTTTTTT |
| 27[84] | 6[82] | AACCATCACCCAAAAATCGGCGCCCGCTTCACATTAATTGCGTT |
| 28[189] | 29[160] | TAACAGGATTAGCAGAGCGAGGATCTAAAGTTTTGTCGTCTCCTCATA |
| 29[119] | 8[112] | GTATAACGTGCTGCTTCTAAATCGGAACCCTAAAGCGAGCACCGAACTA |
| 29[13] | 29[41] | TTTTTTTTTTCTGGCAAGTGTAGCGGTCA |
| 29[147] | 7[160] | AGACAGCTTCCAGAGCTAAACAACTTTCGAAAGGAGATGGTT |
| 29[49] | 31[62] | CGTAACCACCACACCGCCAGAATCCTGAGCAAATTAACCGTT |
| 29[63] | 26[63] | CCGCCGCTGGCGAGAGCGGTCCACGCCAAGGCGGTTTGCGTA |
| 29[70] | 31[83] | GCTTAATGCGCCGCGGATTTTAGACAGGTACTTCTTTGATTA |
| 29[91] | 0[86] | CGCGTACTTACAGGCCACATTGTAACGCCAGGG |
| 3[168] | 9[169] | CTGTAGCTGTTAAAATTCGCAAAACTAGATCGGTGGAGAAACAC |
| 3[189] | 0[182] | ATTAAATAAATTGTAAACGTTGTACCCCGCGCAAC |
| 3[89] | 44[91] | CCCCGGGTATTGATAACAAAGCGGATAAAT |
| 30[189] | 10[154] | TAACAGGATTAGCAGAGCGAGGTGTACCGTAACACTGAGTTCAAGCCCAATCTTG |
| 30[41] | 30[13] | TTTTATAATCAGTGAGGCCTTTTTTTTTT |
| 30[48] | 26[42] | GAAGTGTCGCTGCGAAGCGAAGGCCCTGAGAGAGTCCAGGGTGGTTTTT |
| 31[105] | 27[118] | GTAGAAGCGGGAGCGCTTTGAGGAGCCCTTTTGGGGTCGAGG |
| 31[119] | 10[112] | ACTATCGGCCTTACACTTCCTCGTTAGAATCAGAGAACTCAAAAAGGAA |
| 31[13] | 31[41] | TTTTTTTTTTACCGAGTAAAAGAGTCTGT |
| 31[133] | 14[133] | CACCCTCTTCATTAGATGAACAACTGACGTGTCGAGATTTGT |
| 31[140] | 35[139] | ATTTTCACGCCACCCTCAGAGGAATAGGAGAGGGTAGGCTGA |
| 31[91] | 14[91] | CATCACTAAGAGCAATAAAAATAAAATGCGTCATAGACCATA |
| 31[98] | 36[91] | TGCCTGACGCCAGCCATTGCAGACCTGAAATGCGCTAAAGCAGCAAATC |
| 32[189] | 33[160] | TAACAGGATTAGCAGAGCGAGGACCCTCAGAACCGCCACCCCAGGAGG |
| 32[41] | 32[13] | GAAATGGATTATTTACATTTTTTTTTTTT |
| 32[62] | 36[49] | CATTTTGACGCTCATAATAAACCGAACGTGCAACATATCTTT |
| 32[83] | 36[70] | AAACGCTCATGGAACAACAGAAAAACATGCCAGCAATTGAGG |
| 32[90] | 28[84] | ACAGGAAGTAATAAATTAAAGTACAGGGGACGGGG |

| 33[112] | 32[112] | GTGGCACAGACATATAGCCCGCCGCTGGTAATATCCAGAACA |
| --- | --- | --- |
| 33[13] | 34[13] | TTTTTTTTTTGGCAGATTCACCGATAAAACAGAGTTTTTTTTTT |
| 34[146] | 30[147] | GCCGTCGTGTATCACCGTACTTCAGAACGGGATAGTCGTCAC |
| 34[153] | 7[153] | GATAAGTAGACCAGACAAGAAAGGCTTGTTGGACGAGTCAGA |
| 34[160] | 38[147] | CCAGGCGAGGATTACCTGCCTGTAACAGACAGGAGTGTACTG |
| 34[189] | 33[169] | TAACAGGATTAGCAGAGCGAGGTTTTGCTCAGTATTTAGTACC |
| 34[48] | 31[48] | AACCACCAGCAGAAAGTCACACGACCAGATCGTCTCCATCAC |
| 34[69] | 30[63] | CGCCATTAAAAATAAGGGACATTCTGGCATACCTAGTAGCAAAACGGTA |
| 35[112] | 10[119] | CTCAAATATCAAAGTATTAAGTGATATAGAGGACACCCAAATCAACGCA |
| 35[13] | 36[13] | TTTTTTTTTTGTGAGGCGGTCACTAATAGATTAGTTTTTTTTTT |
| 36[139] | 13[153] | TATTCTGGTAACGGGGTCAGTGCCTTGAATTTCGGGACCTGC |
| 36[189] | 35[169] | TAACAGGATTAGCAGAGCGAGGCAGTTAATGCCCGGATTAGCG |
| 37[14] | 38[7] | TTTTTTTTTAGCCGTCAATAGTTGTTTGGATTATACTTCTTTTTTTTTT |
| 37[35] | 35[48] | ATAATACATTTGAGAGGAGCACTAACAAGTATTAACACCGCC |
| 37[49] | 40[42] | GATTTAGAATTCATCAATATAATATCAAAATTATTTTTCAATTACCTGA |
| 37[70] | 40[63] | ACAAACATCCTGATTATCAGAAAAACAGAAATAAACGCGCAGAGGCGAA |
| 37[77] | 12[75] | ATTCGACAACTCGTAACAGTTGCGGAATTTTAGACTGGATAGCG |
| 37[91] | 14[79] | ATTAAATGGAGCGGAATTATCCGTAGATAATCAAAAATCA |
| 38[189] | 37[169] | TAACAGGATTAGCAGAGCGAGGGCTTTTGATGATTGCCCGTAT |
| 38[62] | 35[69] | TGATGGCAAGTATTAGACTTTAAGGTTATCTAAAAGTGCCACGCTGAGA |
| 39[119] | 13[132] | AGTAACAATTTTGCTTTTAAAAGTTTGAAAACATGATAAATT |
| 39[126] | 18[133] | GTACCTTATCATCGGAAAGAGCATCTGCAGCAATAGGAACGCGGGTAGC |
| 39[133] | 38[126] | TTAGTCTCTGAATTGTAATAAGTTTTAACATTATC |
| 39[147] | 21[153] | TACCGTTCAAAGTATAAAACAGATGGGCAATAATTTTTGTTATTGCGCC |
| 39[6] | 39[34] | TTTTTTTTTTGAATAATGGAAGGGTTAGA |
| 39[91] | 16[79] | TTTCAGGTTGAATACCAAGTTAAATCAAAATATCGCGTTT |
| 39[98] | 13[111] | TTTAACGACCAGAACCTTTGCCCGAACGTATCTGGATTGAAT |
| 4[139] | 20[126] | TCATTTTTTAACCTAAATGGTCAATATAGCTGCAACTAAAGT |
| 4[228] | 5[228] | TTTTTTTTTTGAAGATTGTATAAGCAAAAGCCCCAAAAACAGTTTTTTTTTT |
| 40[189] | 39[172] | TAACAGGATTAGCAGAGCGAGGAACAAATAAATCGCGTCATACATG |
| 40[34] | 40[6] | AAGATGATGAAACAAACATTTTTTTTTTT |
| 41[126] | 44[126] | TCGCTATCAACCTATCCTACAGAGGCTTCAGAGCCGCCACAA |
| 41[147] | 20[147] | TCAGACGGTAATGCTAAAGACCAGCATCGTTAAAGATTCGGT |
| 41[42] | 44[42] | ACAATTTCATTTGACAAAATCATAGGTCTAAATGCAAAAAGC |
| 41[6] | 41[34] | TTTTTTTTTTCAAGAAAACAAAATTAATT |
| 41[63] | 45[69] | TTTTTAATGGAAACAAGAGTCAATAGTGTCGCAAGCACCGGAGAATCGC |
| 42[118] | 47[111] | AATCCTTGAAAACATAGTTAAAATACCGGGCATTTACAAAAGATTAAAC |
| 42[146] | 43[146] | GCATTGACAGGAATTAATTTTCTCCTCAGAACCGCCACCCTC |
| 42[189] | 41[172] | TAACAGGATTAGCAGAGCGAGGCAGAACCACCACTTGATATTCACA |
| 42[34] | 42[6] | CTACCTTTTTAACCTCCGGTTTTTTTTTT |
| 42[97] | 45[90] | AGCTTAGATTAAGAAAAACTTAAGGCGTCCAACAT |
| 43[28] | 38[35] | TATATAACTATATGTGAGAGAACATTTAGCAAAAGACCTACCATCCTGA |
| 43[49] | 39[62] | TGATGCAAATCCAAAATTTATATTACCTTTATTCATGCACGT |
| 43[6] | 44[6] | TTTTTTTTTTCTTAGGTTGGGTATGCGTTATACATTTTTTTTTT |
| 43[70] | 38[77] | ACAAAGAACGCGAGCGCTGAGAGTACATACAAAATGAAATTGATCATAT |
| 43[91] | 40[98] | TTTCAAATATATTTTAGCGATGAGTGAAGATTGCT |

| 44[118] | 40[119] | GGTTTGATTTCATCTTCTGACCCCTTAGTAAATCGGAAACAA |
| --- | --- | --- |
| 44[189] | 17[172] | TAACAGGATTAGCAGAGCGAGGCGGAACCAGAGCTGAGGAAGTTTC |
| 45[112] | 41[118] | TCGAGCCAGTAATAATTTAATCAACAGGAACAAAAAGATTAAGCTTCTG |
| 45[126] | 15[146] | AGTAGCCCCCTTATTAGCGTTCCTCCCTTGAGGACCACTACGAATACAC |
| 45[147] | 20[140] | TGCCATCCGCGTTTATCAGTATGAAACCATCGATAGCAAAATCGCTGAG |
| 45[42] | 48[35] | TCAACAGCGCGCCTTTTACGACGCGAGGCGTTTTA |
| 45[6] | 46[6] | TTTTTTTTTTAATTCTTACCAGGATAAGTCCTGATTTTTTTTTT |
| 46[104] | 12[98] | GTAAAGTATTGCTGGCTCCTTCCGAGCTGTCGACTCGACGTTGTAATAG |
| 46[189] | 19[172] | TAACAGGATTAGCAGAGCGAGGCTTTAGCGTCAGTTGCGGGATCGT |
| 46[41] | 44[28] | GTTTATCAACAATATATAAAGCCAACGCCTGTTTAGTATCAT |
| 46[62] | 44[49] | CAGCTAATGCAGAATAGGGCTTAATTGAATCATAATTACTAG |
| 46[69] | 49[69] | ACATGTTATCAATAGCTTATCCGGTATTTATTTTGCACCCAG |
| 46[83] | 44[70] | GACGACAATAAACACATATTTAACAACGTAAATAAGAATAAA |
| 46[90] | 49[90] | TCCAGACCTTATCACAAGCAAATCAGATTTTATCCTGAATCT |
| 46[97] | 15[104] | AATTCTGGTAATTTAGGCAGAACCGTGTAACCAGACCCGAAAATAGTCA |
| 47[126] | 19[146] | ATCGAGAACAAGAGCACCGTATCATCGGGCAGGGA |
| 47[6] | 48[9] | TTTTTTTTTTACAAGAAAAATATCCCGACTTTTTTTTTTTT |
| 48[111] | 4[93] | AATCATTACCGCGCGCTAACGCCATATATTTAGTTTCCGCTCACAAT |
| 48[189] | 49[169] | TAACAGGATTAGCAGAGCGAGGAGGCCGGAAACGTTACCATTA |
| 49[105] | 23[104] | AGCGTCTAATCCAAATAAGAATGAGTTAGCTGAAATTAACAT |
| 49[112] | 19[125] | TTCCAGATCGTAGGCAAGTACCGCACTCAATATAAGCTCAAC |
| 49[126] | 20[119] | TTGCCAGTTACACAGCCAAGCCGTTTTTATTTTCAGCCTAATACGGTGT |
| 49[147] | 23[146] | CACCAGTATTTGGGAATTAGATTGACGGACAGCTTCCAAAAG |
| 49[154] | 47[172] | AGCACCATCACCAAGCGACAGAATCAAGTTTGC |
| 49[35] | 51[48] | CCTTAAATCAAGATGAATAACATAAAAAAACACCCTGAACAA |
| 49[9] | 50[9] | TTTTTTTTTTGCGGGAGGTGCGCATTAGTTTTTTTTTT |
| 5[93] | 10[98] | TCCACACAACATAAGTTGGCAACTAAGCATAGT |
| 50[189] | 51[169] | TAACAGGATTAGCAGAGCGAGGACCGTCACCGACAGGTGAATT |
| 50[34] | 47[41] | CAGGGAATTTGAAGGCGAACCATATCCCATCCTAA |
| 50[69] | 53[62] | AAATAGCAGCCTTTGGGTAATTGAGCGCGAAACCGAGGAAACAAAATAC |
| 50[90] | 53[83] | TTTGTTTAACGTCAAGAGAGATAACCCAGCCGAACAAAGTTACAACATA |
| 51[119] | 8[119] | AGCAAGAATATTTTAGGCAAGGCCTGGGCAGGTCAAATAAAA |
| 51[126] | 50[119] | AACAATGAAATATAGCAAATAAACAGCCATATTAT |
| 51[70] | 47[83] | TAATATCAAAATGACTACAATATAGAAGATCGGCTGTCTTTC |
| 51[9] | 52[9] | TTTTTTTTTTACGGGAGAATGGCATGATTTTTTTTTTT |
| 51[91] | 47[104] | CAAGAATACGATTTTACCAACCCAATAGTTCCAAGAACGGGT |
| 52[189] | 53[169] | TAACAGGATTAGCAGAGCGAGGGGCGACATTCAACAAAGACAA |
| 52[48] | 26[35] | TAACGGAATACCCAGCAGTATCAACGCATAAATGCCTTTTCA |
| 52[55] | 47[62] | GCAATAAAGTCAGAACAGAGATAGTTGCCTAAGAAGCATGTAGAAACCA |
| 53[119] | 27[139] | TTTTGTCATAAAGCATTCAACACAAAGGTGCCGTAAAGCATTCTGTATG |
| 53[126] | 52[119] | ACAATCAATAGAAAAAGCAATAGCTATCTTACCGA |
| 53[9] | 51[34] | TTTTTTTTTTTAAGACTCCTTATTACAAAGAACTTAACTG |
| 6[104] | 30[91] | AGCTAACTTCCAGTAACATTATATGGTTTAAACAGGAGGCCG |
| 6[118] | 17[118] | GTGCCTAGGAAGCAGTGTGAAGTAATCAGAGTACCCAAACTC |
| 6[146] | 22[126] | TTTTAAGAACTGAGTCTGGAAGCAAAGAATTAGCTGAACCTGTTTAGCT |
| 6[160] | 18[154] | ATTACCTAATCGTATTAAATTCGCGTCTCGAAAGA |

| 6[189] | 7[169] | TAACAGGATTAGCAGAGCGAGGTAATCATTGTGATAATTTCAA |
| --- | --- | --- |
| 7[82] | 24[91] | GCGCTCACTCAACGCGAAGGCCGGAGACAGGGTTGTA |
| 8[132] | 24[126] | CTACGTTTTGCCTGGAGATCTCGTTCTAGCTGATAGGCAAAATTAAGCA |
| 8[139] | 15[139] | GAAAAATTCATTCACAGCTGGGACGACGAACCGTGGCAAAAG |
| 8[160] | 15[160] | ATTGGGCCCCTGACCGGGCCTAGGAAGATGGTGTACTCATCT |
| 8[189] | 29[169] | TAACAGGATTAGCAGAGCGAGGAACGAGTAGTAAGTTAGCGTA |
| 9[112] | 13[118] | ACATAACGCTGCAAGACGGCCAGTGCGTGAAGAAGTTTTGCCCCCCCTC |
| 9[196] | 2[168] | TAACAGGATTAGCAGAGCGAGGTGTTGGGGCTTTCCCCGTAATGGGATAG |
| 9[75] | 10[75] | TTGAGATTTCATAACCCT |

**Wheel pore**

Start End Sequence

0[147] 5[144] TTTTTTTTTTGTTTAGTATCATACCTCCGGCTTTTTTTTTT

1[112] 4[105] GGAATCAATACCGACCGTGTGAAACTTTTGTAAAT

1[54] 5[76] TAACAACGCCAACAAATGAGAATTTACGCTGAGAAGA

10[117] 11[92] TAACAGGATTAGCAGAGCGAGGGCGAATTATTCAAATCGCGCA

100[117] 100[77] TAACAGGATTAGCAGAGCGAGGTCAATAGATAATACATTTG

101[23] 102[23] TTTTTTTTTTACAGACAATCGCCATTAATTTTTTTTTT

101[49] 100[49] AATGGCTATTAGTCTTTAAAGAGAAGTATTCTGACCTGAAAG

102[117] 101[95] TAACAGGATTAGCAGAGCGAGGGAGGAAGGTTATAGATTAGAGCCG

102[69] 99[69] GGTGCGCGAACTGATAAGAGCGGGAGCTTGAGAAGACTTTAC

102[83] 104[70] CTAAAATAGGCCGATATAACGTGCTTTCGCTACAGATCTGGT

103[23] 104[23] TTTTTTTTTTAAATACCGAGCCTGCAACTTTTTTTTTT

103[42] 100[23] ACGAACCAAAACATATTTTTGCGTAAGAATACGTGGCTTTTTTTTTT

104[117] 103[95] TAACAGGATTAGCAGAGCGAGGTATCAAACCCTCTTGAAAGGAATT

105[23] 58[23] TTTTTTTTTTAGTGCCACGATTGTGTCGTTTTTTTTTT

105[70] 27[76] AAAGCATCACCTTGCGTGGCGCGCTAGGAAATAAATCCTCCTTGAGGCA

106[117] 65[92] TAACAGGATTAGCAGAGCGAGGTTCGAGCTTCAACGGCAAAAT

106[62] 68[56] ACTCCAACTAAGAGGAAGCCCGCCCTGAGAGAGAA

106[76] 65[62] CAGACCGGAAGCAAAATTGCTCCTTTTTCCCGAGATAGGGTTGAGTGCTTACCTTT

106[83] 111[91] AGCGAACTTCAAATGCAAAGCCAGGTCTTGCTTTACGTCATAAATATTCA

107[20] 108[20] TTTTTTTTTTTGCTCATTCAGTACGAGTAGTAAATTTTTTTTTT

107[42] 65[55] GAATAAGCTAAAGTAGCTCAACATGTTTCGGATGGTAGAGAG

107[49] 106[20] GCTTGCCCAGGTCAGGAATCAACGTAACAAAGCTTTTTTTTTT

108[117] 107[92] TAACAGGATTAGCAGAGCGAGGTTATAGTCAGAAATCGCGTTT

109[20] 110[20] TTTTTTTTTTTTGGGCTTGAGAACCTTATGCGATTTTTTTTTTT

109[56] 112[56] CTATGACCATAAATTTTGCGTATTGGCATCAATAATTTAGAC

11[27] 14[27] TTTTTTTTTTACAAAATTAATTGAGTTTCGTCACTTTTTTTTTT

11[63] 13[95] GATAGCAAGCCCAAGAACCGCATATGTGAGGGTTTAGTACCGCCACC

110[117] 109[92] TAACAGGATTAGCAGAGCGAGGTATCCCCCTCAAATTACCCTGA

110[76] 112[63] AACAGTTCAGAAAACGAGATTTAGCGTCCAATACTTAAAATG

111[20] 68[49] TTTTTTTTTTTTTAAGAACTGGCTCATTATACCAGAAATCATTGTGAATTTGGTTTAGAACGAGTATAACAGTTGATTTATGCAA

112[117] 71[92] TAACAGGATTAGCAGAGCGAGGAGAAGTTTTGCCCTGCATTAA

113[20] 72[20] TTTTTTTTTTTACGTTAATAAAACGAACTAACGGCATGGTCAGGACGTTGCATTTCGCATTTGGGGCGCGAGCTGATTTTTTTTTT

113[56] 73[76] GACGACGATAAAAACATAACCATGGTGCCTAATGA

114[117] 73[92] TAACAGGATTAGCAGAGCGAGGACGAGGCATAGTAACTCACAT

115[20] 76[42] TTTTTTTTTTATTCATCAGTTGAGATTTACTAAATC

115[56] 77[76] CACATTCAACTAATTAGCTGTTTCCTAGCGGGAGAAGAGCCTCCTCACA

116[117] 117[92] TAACAGGATTAGCAGAGCGAGGTTCAGCAAATCGTACACTGGT

116[83] 86[63] TTAACGGCAGTACAGCGCCATTAGCTCGTCATAAATCACGACGTTGTAC

117[27] 118[27] TTTTTTTTTTGATAGACTTTCTAGACGCAGAAACTTTTTTTTTT

117[49] 84[49] CCGTGGTTCGATGATGAGAGTCTGGAGCAATTTTGAGAGATC

118[117] 119[92] TAACAGGATTAGCAGAGCGAGGCCTGCGGCTGGTGGTCACTGT

118[83] 119[76] AATGGGTAAAGGTTTCTTTGCATGCGGTTGCGGTA

119[27] 120[27] TTTTTTTTTTAGCGGATCAAACGGCGGTTGTGTATTTTTTTTTT

12[117] 17[95] TAACAGGATTAGCAGAGCGAGGCTCAGAACCGCCACGTTAGTAAAT

12[62] 4[51] AGAGCCACCACCATGGAAACAGTACATATGAGAGAAGGATTAATAAGTGCCGTCGATAAACAGTTAAT

12[69] 44[49] CACCCTCAAAGTTTACAACTTTGAATAAAAAGAAATTAGGAGCCTTTAA

12[83] 8[70] ACCCTCAGTTACAATTTCAATTACCTGAGCTATTAAATAACC

120[117] 121[92] TAACAGGATTAGCAGAGCGAGGTGCAGGCGCTTTCAGCGGGGT

120[83] 121[83] CGCACTCAATCCGCCGGGCTTAAAGCCGCAGGTGTCCAGCAT

121[27] 90[27] TTTTTTTTTTCATCGACATAAAAAAATCCATGAAGGTAAGCAAAAATTCGCATTAAATTTTTTTTTTTTTTT

121[56] 88[49] CGTAAAAGCCGCCAGCAGTTGTTAAATTTCAGAAA

122[117] 123[92] TAACAGGATTAGCAGAGCGAGGCCACGCAACCAGGGTGGTGCC

122[83] 119[83] CTTACGGCTGGACAAATGTTGGGAAGGGTTACGCCTGAGCCG

123[27] 92[27] TTTTTTTTTTGATGCTGATTGCCGTTCCGGGCAGCCCATTTTTCGTCTGGCCTTCCTGTAGCTTTTTTTTTT

124[117] 125[92] TAACAGGATTAGCAGAGCGAGGAGCAGCAACCGCGCTGGTCTG

125[27] 93[55] TTTTTTTTTTACATCCTCATAACGGAACGCCGTCGGACATTAA

125[63] 95[76] CTTGTAGAACGTCATGAGGGGACGACAGGATAGGTCACGTTG

13[23] 8[23] TTTTTTTTTTAATTACCTTCGGGGTTTTTTTTTTTTTT

14[117] 15[92] TAACAGGATTAGCAGAGCGAGGCGCCTGATTGCTCAATAACGG

14[55] 7[62] TAACACTACCTCATTTTCAGGAAGATGATGAAACATGAGACTCCTCATA

15[27] 42[27] TTTTTTTTTTCAGTACAAACTATTTCACGTTGAATTTTTTTTTT

15[49] 91[62] CAGCGGAGTGAGAATGTACCGATGTGAGTAATTCGTAACCAA

16[117] 45[95] TAACAGGATTAGCAGAGCGAGGGAATTTTCTGTACCTACCATATCA

16[83] 92[63] TGGGATTGGAGAAATTGAATACTCAGGAGGCACCGCTTCTCATCAAAAA

17[23] 12[23] TTTTTTTTTTAGACAGCCCTTTCATTTGTTTTTTTTTT

17[42] 44[23] TCATAGTGCCTGTACCGACAATTGTATCGGTTTATCATTTTTTTTTT

17[70] 52[70] TGTCGTCCCTTGCTTAGTAATACCGTTGTCAGTGAAGAATCC

18[117] 18[70] TAACAGGATTAGCAGAGCGAGGTACAGGAGTGTACTGGTAATAAGTGG

19[24] 18[24] TTTTTTTTTTGATTAAGACTCCTCACCCAAAAGAACTGGCATTTTTTTTT

19[56] 2[58] AAGGGCGTATGGTTGGTGGCAACATATAGAACAAAGTTAC

19[63] 22[49] ACATTCAAAACGCAATAATAACGGAATAAAGACAAGGTAAAT

19[70] 21[92] ACAGCGTCATACATTCACAATCGCAAAGACACCACGG

2[104] 3[104] ATAAATAAGGCGTTAAGAAAAAGCAAGACAAAGAACGCGAGA

2[149] 3[150] TTTTTTTTTGACCTAAATTTAATGAGTTAATTTCATCTTCTTTTTTTTT

20[117] 19[95] TAACAGGATTAGCAGAGCGAGGAAGTTTATTTTGGGCTTTTGATGA

20[76] 63[76] CAATAGACACCATTCGATAGCGCCACCCACCAGAGGGAACAA

21[20] 20[49] TTTTTTTTTTGCAAACGTAGAAAATACATACATAAATACCAGC

22[117] 22[70] TAACAGGATTAGCAGAGCGAGGTGTCTCTGAATTTACCGTTCCAGTACG

23[24] 22[24] TTTTTTTTTCATTAAAGGTGAATAAATTGACGGAAATTATTTTTTTTTTT

23[49] 61[69] TCAAGTTTCGGCATATCTTTTAGCCGGAGAACTGACCGGGCGATGGCCC

23[56] 20[63] TGCCTTTTAATCAGAAAATCACCAGTAGAAATTCA

23[70] 25[92] GAATTAAAGCCAGAAAACCATACCATTAGCAAGGCCG

24[117] 23[95] TAACAGGATTAGCAGAGCGAGGACGTCACCAATGATGGAAAGCGCA

25[24] 20[24] TTTTTTTGAGCCATTTGGGAATTAGGCTATTACGCAGTATGTTATTTTTT

25[49] 62[42] AGCCAGCTAGCGACAGAGCCGATCAAAACTGGCTGCTTTGAAAGAGGAC

26[117] 57[95] TAACAGGATTAGCAGAGCGAGGATATTCACAAACGCGCTGGCAAGT

27[23] 28[20] TTTTTTTTTTCTTATTAGCAACCAGAGCCACTTTTTTTTTT

28[117] 27[92] TAACAGGATTAGCAGAGCGAGGAGCCGCCACCAGCGATTGGCC

28[69] 67[76] CCGCCGCCAGCATACCACCCTATTTTTGTATTGCAGCAAGCGGTCCACG

29[20] 66[20] TTTTTTTTTTCACCGGAACCGCCTTAATTGCTGATTTTTTTTTT

29[42] 24[20] CTCCCTCAGTATCACCGTCACCGACTTTTTTTTTTT

3[112] 1[147] TTCAAATATATTTTGTTTGAATAATTACTAGAAAAAGCCTTTTTTTTTTT

3[58] 13[69] CAGAAGGAAACCGCCCGTAGAGGGTTTTGCTTCAATCAAT

30[117] 7[92] TAACAGGATTAGCAGAGCGAGGTAATAAGAGCAAAAGCCCTCGCTCAAGTGAATTAAAACATTTCCCTTAG

30[147] 4[112] TAACAGGATTAGCAGAGCGAGGAAACACCTTCTTACGTCTGAGAGACTACAACTATA

31[20] 34[20] TTTTTTTTTTAGAATATAAAGTATTACCGCGCCCTTTTTTTTTT

31[42] 30[20] ACCGACAAAAGGTAAGAGGCATTTTCGAGCCAGTAATAAGTTTTTTTTTT

31[56] 78[56] ATATCAGTTTATTTTTACAGAACGTCAAAGCGAACTTTATTT

32[117] 33[92] TAACAGGATTAGCAGAGCGAGGGATAAGTCCTGATTATCAACA

32[69] 21[76] GAGGGTAGCTAATGCAGAACGGCTTAATTAGCTATGATAGCCAAAGAAA

32[83] 30[56] ACAAGAAAAATACAACCCACATGAAATAGCTGTAATTTAGGC

33[27] 32[56] TTTTTTTTTTGACGACGACAATAAACAACATGTTCAATTGAGC

34[117] 31[92] TAACAGGATTAGCAGAGCGAGGAGTACCGCACTCAGTTAAGCC

34[83] 79[91] ATCGAGAAAGAACGTAAGAAATAAACAGCCTGTTCGTCATACCGGGGGTT

35[21] 36[27] TTTTTTTTTAATAGCAAGCAAATCAGTAGGGAAGCGCATTTTTTTTTTTT

36[117] 37[92] TAACAGGATTAGCAGAGCGAGGTGTAGAAACCAAAATTTACGA

37[27] 32[27] TTTTTTTTTTAGACGGGAGAATTAACTGAGCTAAAGTAATTCTGTCCATTTTTTTTTT

37[56] 80[49] ACACCCTATAACATTTTTGCACGACTTGCACCATCTAGGTAAAGATTCA

37[63] 81[83] GAACAAAGTATATCCCATCCTTCAATAATATCCTGCTTTCCATGTCACT

38[117] 35[92] TAACAGGATTAGCAGAGCGAGGTCCCAATCCAAAGGTATTAAA

38[69] 31[69] TTGTTTAGACCTTATCATTCCACAAGCAAGCCGTTAGAGATA

38[76] 75[69] CGATTTTTATCCGCTGTAAAGCCTGGTAACATCCAAGGTGTGAAATTGT

39[20] 78[20] TTTTTTTTTTGGTATTCTAAGAAAATTTTTAGAATTTTTTTTTT

39[49] 72[49] GCGTTTTAAATGAAAAGCCTCATAAATCCTAATAGATATTTT

4[144] 0[112] TTTTTTTTTTTTAGGTTGGGTTATATCTTTTTAATGCGTTATACAAA

4[76] 31[83] TAACAGTGATTTAACGGGGTCGTAAGCACTTACCGGAAACAAAGAATTG

40[117] 41[92] TAACAGGATTAGCAGAGCGAGGGCTAACGAGCGTAATCTTACC

41[27] 84[27] TTTTTTTTTTAAATCAAGATTATACAAAGGCTATTTTTTTTTTT

41[49] 34[42] GTTGCTAAAAAACAGCAGCCTTCATCGTAGGAATC

41[63] 85[83] CCCAGCTTCACCGGAAAAAACGACGGCCAGTGCCA

42[117] 43[92] TAACAGGATTAGCAGAGCGAGGCAGTAACAGTACCAGATGAAT

42[76] 44[70] ATACTAAAGGAATTGTAGATTTTCAGGTCAGAAAT

42[83] 124[70] CTTTTACGTGTAGAGCCAGTTGCGTGGTAAGAATGCCAACTT

43[27] 42[56] TTTTTTTTTTAATCTCCAAAAAAAAGGCTCCAAAGCGCGAATA

44[117] 43[83] TAACAGGATTAGCAGAGCGAGGAAATTATTTGCACGTAAAATTAACGT

45[23] 16[23] TTTTTTTTTTATAGTTGCGGCATTCCACTTTTTTTTTT

45[49] 17[55] TGACAACAACCATCTCAACAGTTTCAACTAGCGTA

45[70] 17[83] TGGAAGGTTGTTTGACTTCCTGATTATCCTATCGGTTTCCAG

46[117] 45[83] TAACAGGATTAGCAGAGCGAGGTTCATCAATATAATCCTGAGTTAGAA

46[69] 97[62] GATTATACTTCGCCCACGCATCCAGCCAACATTTTTTGGCAGATTCAGT

47[24] 46[24] TTTTTTTTTCGCTGAGGCTTGCACGAACCGATATATTCGGTTTTTTTTTT

48[117] 47[95] TAACAGGATTAGCAGAGCGAGGGAAGAACTCAAAAGATGATGGCAA

48[55] 47[69] CAATATTCACCCTCAGCAGTTCTTTGATGGTAATAAGGAAAA

49[24] 48[24] TTTTTTTTTGCTTTTGCGGGATCGTACGGGAGTTAAAGGCCTTTTTTTTT

5[51] 0[54] GCCCCCTGCCTACGCCATATT

50[117] 97[95] TAACAGGATTAGCAGAGCGAGGCTGTCCATCACGAAAGAAACCACC

50[62] 51[69] ACCGAAAGACAGCATTGAGGACTAAATTTTTATAA

50[69] 17[62] TAGCAATAACATTATCATGGAAATACCTTTGCAACTCCAGAAACGATCT

51[23] 96[23] TTTTTTTTTTAACGGCTACAGAGGCTTCGGAACATTTACAGACGCTCAATCGTCTGTTTTTTTTTT

52[117] 99[95] TAACAGGATTAGCAGAGCGAGGTAGACAGGAACGCTCGTATTAAAT

52[48] 98[23] AGGAAGTCAACAGACCAGTAATAAAAGGGATTTTTTTTTT

52[62] 103[69] TGGACTTTTTCATGATGCCACTAGCCCTACCAGCAGAAGATAAAACAGT

53[23] 54[23] TTTTTTTTTTACGGGTAAAAAACGAAAGTTTTTTTTTT

53[70] 55[83] AAACAGGATCTTTATGGCAAATCAACAGAATCAATGGCGCGT

54[117] 53[95] TAACAGGATTAGCAGAGCGAGGTTGACGAGCACGTTAAAGGGATTT

55[23] 56[23] TTTTTTTTTTAGGCAAAAGCGATTATACTTTTTTTTTT

55[49] 58[49] TAAAACATCAGTATCCAGCAGCAAATGAGATTGTATCATCGC

55[56] 54[49] CTGCTTAATGCGCCCTCGTTAGAATCCGAAGGCAC

56[117] 55[95] TAACAGGATTAGCAGAGCGAGGGTAGCGGTCACGACTATGGTTGCT

56[69] 26[56] CACACCCGCCGCCATCTTTGATACAACGGAGATAAGTTTTCA

56[83] 59[83] CTGCGCGTAACCACGAGCGGGAGAAAGGAGCCCCCGATTTAG

57[23] 26[23] TTTTTTTTTTCAAGCGCGACATAGCCCCTTTTTTTTTT

57[42] 53[48] AACAAAGCCCCCAGAATACACCAACCTAATACGTA

58[117] 105[95] TAACAGGATTAGCAGAGCGAGGAAAGCCGGCGAACTGAACCTCAAA

59[23] 60[23] TTTTTTTTTTAAATCCGCGGCAGACGGTTTTTTTTTTT

59[49] 104[56] CCATGTTACAACCCTAAAGGGAAGGGAAAAAATCTCAGAGGTGAGGCGG

6[117] 9[95] TAACAGGATTAGCAGAGCGAGGCCTTGTATCAAACAAATCCAATCGTGCCGGAATAGGTG

6[62] 6[27] GGAACCTATTATTCTGAAACATGAAATTTTTTTTTT

60[117] 59[95] TAACAGGATTAGCAGAGCGAGGGTTTTTTGGGGTAGCTTGACGGGG

60[69] 57[69] GCACTAAATCGGTTCATTGACAGGAGGTGTAGCGCGCGAAAG

60[76] 65[83] CCGTAAAACTACGTGAACCATGTCAAAGGAGTCCAAGAATAGCCGAAAT

60[83] 68[77] CGAGGTGGGTCAGAAACCACCTCAGAGCTGATGGTCTGGTTTCCCTTCA

61[23] 62[20] TTTTTTTTTTCAATCATAAAGATGAACGGTGTTTTTTTTTT

61[42] 104[42] GGGAACCACGAGGCACCTGCTCTGATAACTGAGAGTAACACC

62[117] 61[95] TAACAGGATTAGCAGAGCGAGGGTGGACTCCAACCACCCAAATCAA

62[76] 63[69] GGCGAAAAACCGTCTATCAAAACTTGTTCCAGTTT

63[20] 64[20] TTTTTTTTTTTACAGACCAGGCCTTGACAAGAACTTTTTTTTTT

64[117] 63[92] TAACAGGATTAGCAGAGCGAGGTTATAAATCAAACTATTAAAG

64[55] 26[42] TCATCAAGAGTAATGCATAGGTCACCGGGTTTGCCTTTCGGT

65[20] 66[42] TTTTTTTTTTCGGATATTCATTACCCAATCTTAGAG

66[117] 29[92] TAACAGGATTAGCAGAGCGAGGGAAAATCCTGTTCACCACCCT

66[76] 22[56] GGTGATAAGAGGTCCAGAACCAGCACCGAGCGTCAATTGAGGGAGGGAA

67[20] 68[20] TTTTTTTTTTATATAATGCTGTACGGTGTCTGGATTTTTTTTTT

68[117] 67[92] TAACAGGATTAGCAGAGCGAGGAACAGCTGATTGGCCCCAGCA

69[20] 70[20] TTTTTTTTTTAGTTTCATTCCATAGATTTAGTTTTTTTTTTTTT

69[56] 107[76] CCGCGCCAGGGTGGTTTTTCTCCGCCTGGAAAGAC

69[77] 113[92] TTTCACCGCGGGGAGTGCCAGAGAGGGGAGCGAGAGGCTTTTGC

7[27] 10[27] TTTTTTTTTTGTATTAAGAGGCAACATCAAGAAATTTTTTTTTT

7[77] 10[63] ATTAATTAGCGATAGCTTAGATTAAGTCAATCGTCGCAAAAG

70[117] 69[92] TAACAGGATTAGCAGAGCGAGGATCGGCCAACGCAGTGAGACG

71[20] 112[20] TTTTTTTTTTGACCATTAGATAGGAAGAAAAATCTTTTTTTTTT

71[49] 108[42] CAAATGGATTCTGCATTTCAACGAGAAACACCAGA

71[63] 108[56] CCGGGAAACCTGTCGAGGCGGCAAAAATGGATTGCATCAAAAAGATTGA

72[117] 115[92] TAACAGGATTAGCAGAGCGAGGTTGCGTTGCGCTCACTGCCGTGAGCTAAGAGCACATAACGCCAAAAGGA

72[76] 114[56] CGCTTTCCAGTCTGTTTAGCTTAGTAGCCTCGTTT

73[20] 114[20] TTTTTTTTTTAAAGGTGGCATCACAGGTAGAAAGTTTTTTTTTT

73[42] 74[20] AATTCTAATACAGGCAAGGCAAAGAATTTTTTTTTT

74[117] 77[91] TAACAGGATTAGCAGAGCGAGGACGAGCCGGAAGCATAAAGTCACAATCGTAATCGTTGAGGATCCCCGG

75[20] 76[20] TTTTTTTTTTTTAGCAAAATTAGGTTGTACCAAATTTTTTTTTT

75[42] 38[20] AGCAATAAAATATAGAAGGCTTATCCTTTTTTTTTT

76[117] 75[92] TAACAGGATTAGCAGAGCGAGGCGAGCTCGAATTTCCACACAA

76[76] 111[76] ATGGTCAGCAGATAACACTATCCAAAATGTAATAGGCGGAAT

77[20] 40[27] TTTTTTTTTTAACATTATGACCCTGTAATAGGATAAACGCGAGTTTTGAAGCCTTTTTTTTTTTT

78[117] 39[92] TAACAGGATTAGCAGAGCGAGGCCAGCACGCGTGCCATATTAT

79[21] 80[27] TTTTTTTTTCCCTCATATATTTTAAATGAAAGGGTGAGAATTTTTTTTTT

79[49] 114[42] CAATGCCCAACGCAACTTTTGCATAAAGGGAATACACAACAACATTATT

79[56] 39[76] TGAGTAATGGCCGTTTTCACGTTCGCGTCCGTGCCCTCCAGTTACAAAA

8[117] 8[77] TAACAGGATTAGCAGAGCGAGGTATCACCGTACTCAGGATG

80[117] 81[92] TAACAGGATTAGCAGAGCGAGGACTCTGTGGTGCGCGCGCCTG

80[83] 40[63] TGCGGCCAGAATGCGGCGGTGAAGATCCAGCGCAGGAGCCTAATTTGCC

81[27] 82[27] TTTTTTTTTTAGGCCGGAGACATCTAGCTGATAATTTTTTTTTT

82[117] 83[92] TAACAGGATTAGCAGAGCGAGGGCCAGCGGTGCCGGGTTACCT

83[27] 116[27] TTTTTTTTTTATTAATGCCGGAGAATTTGTGAGATTTTTTTTTT

83[49] 40[49] GAGGGTAGCTATACTGATATTCAACCGTGTCAAATCGGGAGG

83[63] 36[63] AATCGGCGAAACTCGATAACCACAATTTTCGGCTGTCTTTGA

84[117] 85[92] TAACAGGATTAGCAGAGCGAGGTGGAGCCGCCACAGCTTTCAG

84[83] 82[63] GGGAACGAGATGCCGGTGCCCCCTGCATCAGACTA

85[27] 86[27] TTTTTTTTTTCAGGTCATTGCCACGGTAATCGTATTTTTTTTTT

86[117] 87[92] TAACAGGATTAGCAGAGCGAGGAACGCCAGGGTTATTAAGTTG

87[27] 88[27] TTTTTTTTTTAAACTAGCATGTAGCCCCAAAAACTTTTTTTTTT

87[49] 116[49] CAATCATATGTACCAAGAGAAGAAGGGAGTTTACCAGTCCCG

87[63] 117[83] CCGGGGATGTGCTGCAAGGCGTTCCCAGCATCCCT

88[117] 89[92] TAACAGGATTAGCAGAGCGAGGGCCTCTTCGCTACGATCGGTG

88[76] 123[83] AGCTGGCGAAAGGGAATTGTAGGCGGCCCGGTCCGTTGGCAGCACCGTC

89[27] 122[27] TTTTTTTTTTAGGAAGATTGTAGTAAAGTTAAACTTTTTTTTTT

9[23] 12[42] TTTTTTTTTTGCTCAGTACCAGGCGGGGATTAGTTTTAATTTAACAA

9[70] 33[83] GATATAAGTATAGCCCTTGAGGTCAATACAGTAGGCGCCTGT

9[98] 1[104] TAACAGGATTAGCAGAGCGAGGGCTGATGATCATAGCAGTATAAAGCCAATTTTAAATAAGAAT

90[117] 91[92] TAACAGGATTAGCAGAGCGAGGCATTCGCCATTCCAGGCAAAG

90[62] 118[49] TTTGTTAATATTTATTGATAATCTGCTCTCTCACGGAAAAAG

91[27] 124[27] TTTTTTTTTTGTTAAATCAGCTTCCGGCCAGAGCTTTTTTTTTT

91[70] 124[63] GCGGTGCCGGAAACAGGCTGCGCAACCGTTAATATTAGGAACTTCGTCT

92[117] 93[92] TAACAGGATTAGCAGAGCGAGGAGCCAGCTTTCCAGATCGCAC

93[27] 94[27] TTTTTTTTTTCAGCTTTCATCAATTCTCCGTGGGTTTTTTTTTT

93[63] 16[70] CGGACAGTATCGGCCCAATAGGAACCCATAGAAAGGAACACGTTGCTAA

94[117] 95[92] TAACAGGATTAGCAGAGCGAGGAACCGTGCATCTTGGGCGCAT

95[27] 42[49] TTTTTTTTTTAACAAACGGCGGATTGACCATAATTT

95[56] 122[56] GTAATGGTAACAACTGCCGGACGTCGCTGCAAACGTTTAGTG

96[117] 96[70] TAACAGGATTAGCAGAGCGAGGAGAAGGAGCGGAATTATCATCATAGC

97[23] 50[23] TTTTTTTTTTAAATGGATTGAGGGTAGCTTTTTTTTTT

97[70] 101[83] TCATTTTAATTTTAAAACAATAGGATTTCACTAACAACTAAT

97[77] 49[95] GCGGAACCAAATTAAACATCACTTGCCTGAGTA

98[117] 98[77] TAACAGGATTAGCAGAGCGAGGCCTTTGCCCGAACGTTATT

98[69] 99[62] AAAGTTTGACCAGTCACACGAGATAGAACCCTTAG

99[23] 52[23] TTTTTTTTTTCATTCTGGCTTCCATTAATTTTTTTTTT

99[77] 51[95] TCGACAAGTACGCCGGCCACCGAGTAAAAGAGT

| **T pore** |  | |
| --- | --- | --- |
| Start | End | Sequence |
| 0[199] | 0[168] | TTTTTTTTTTTTTTTTCACGCTGCCTGTTTGA |
| 0[39] | 0[8] | GAAAAACCGTCTATCATTTTTTTTTTTTTTTT |
| 0[47] | 2[15] | CAAAGGGCCGTGAACCAAGGGAAGAAAGCGAAAGGTTTTTTTTTTTTTT |
| 1[112] | 3[135] | TTCACCAGTGAGACGGGCAACAGCAAATCAAATTAATTGC |
| 1[144] | 0[160] | CTTCACCGCCTGGCCCTGAGAGATGGTGGT |
| 1[168] | 1[199] | GTTGCAGCAAGCGGTCTTTTTTTTTTTTTTTT |
| 1[55] | 3[71] | CAAATCAAGTTTTTTGGTTAAAGAAACGCTGCG |
| 1[72] | 7[71] | GGTCGAGGCGGGGAAAGCCGGCGAAATCCTGAGTATAACGCAGAACAA |
| 1[8] | 1[39] | TTTTTTTTTTTTTTTTGGGCGATGGCCCACTA |
| 1[80] | 3[103] | TGCCGTAAAGCACTAAATCGGAACGTGTTGTTTAATGCGC |
| 10[111] | 15[103] | GGATATGGCTATTAGAATACATTTCTAAAGCATTCCTGA |
| 10[135] | 15[135] | AGCTTTCTGACGACAGCAACTGTTAATATAAT |
| 10[194] | 10[160] | TTTTTTTTTTTTTTTTGGTTTTCCCAGTCACGAC |
| 10[71] | 15[71] | CTAAAACATAACAACTCAAACCCTGAAACCAC |
| 100[109] | 51[87] | TAACAGGATTAGCAGAGCGAGGATTTGGGAATAAAGCCTCAGAGTGCCGGAG |
| 11[13] | 12[40] | TTTTTTTTTTTTTTTTAAGATAAAACAGAGGTGAGCAGTTGGC |
| 11[160] | 11[194] | TAAGTTGGGTAACGCCAGTTTTTTTTTTTTTTTT |
| 12[199] | 12[168] | TTTTTTTTTTTTTTTTTCTGGTGCCGGAAACC |
| 13[112] | 8[120] | GCCAGTTTGAGGGGACCAGGAGAATTACGCCATATGATAC |
| 13[144] | 8[152] | TCAGGAAGATCGCACTGTTGTAAAAAGGCGATCTAATCTA |
| 13[16] | 12[15] | TTTTTTTTGGAATTGAGGAAGGTTAAATCAACAGTTGAAATTTTTTTTT |
| 13[160] | 15[194] | CCAGCCAAGGCAAAACCCGTCGGATTCTCCGTTTTTTTTTTTTTTTTT |
| 13[168] | 13[199] | GCTTTCCGGCACCGCTTTTTTTTTTTTTTTTT |
| 13[40] | 14[13] | ATCTAAAACGAACGTTATTAATTTTAATTTTTTTTTTTTTTTT |
| 13[56] | 8[56] | AGGAGCACTCGCCATTGTATTAACTATTTACA |
| 13[88] | 8[88] | CAATAGATTCTTTAATAGAGCCAGTACATTTT |

| 14[111] | 17[111] | GTGCGGATTTAGAAGGAATCTTACCAACGC |
| --- | --- | --- |
| 14[135] | 19[135] | GGTGTAGAAGAGCCTATGTTTGGAAACAGGAA |
| 14[194] | 16[160] | TTTTTTTTTTTTTTTTGGGAACAAACGGCGGATTTTTAACCGGCCTTC |
| 14[71] | 19[71] | CAACTCGTTGCTATTTCAAAAGAACCTTAGAA |
| 15[13] | 16[40] | TTTTTTTTTTTTTTTTAAGTTTGAGTAACATTATCATCAAGAA |
| 16[102] | 13[87] | AGAGGCGAATTATTCATTTCAATGCGGAATTTTTACAAAAGAGCCGT |
| 16[199] | 16[168] | TTTTTTTTTTTTTTTTAAAATAATTCGCGTCT |
| 17[112] | 12[120] | TAACGAGCGTCTTTCCTGGGCGCAAATTCATCGGGAAGGG |
| 17[144] | 12[152] | GTTACTCAGCTCATTTGACCGTAAGAGTAACAGCGCCATT |
| 17[16] | 16[15] | TTTTTTTTTTTAACAATTTCATTTAACAAAATTAATTACATTTTTTTTT |
| 17[168] | 17[199] | AATAGGAACGCCATCATTTTTTTTTTTTTTTT |
| 17[40] | 18[13] | GAATTACCATATGTGAGTGAATAACCTTTTTTTTTTTTTTTTT |
| 17[55] | 12[56] | AATGGAAACATTAAATCGGAACAAACAATCAAT |
| 17[88] | 12[88] | TTTATCCTTATTAGACATCATCATATCACCTT |
| 18[100] | 17[87] | CTTGCGGGAGGTTTTGAAGCCGCTACAAT |
| 18[194] | 18[160] | TTTTTTTTTTTTTTTTGTTAAAATTCGCATTAAA |
| 18[71] | 23[71] | AGATTAGTATTCTAAGAATTTAATTTGAGAAT |
| 19[115] | 16[110] | GAAAAGCCCCAAATTATACTTCTGAATAATG |
| 19[13] | 20[40] | TTTTTTTTTTTTTTTTTGCTTCTGTAAATCGTCGCCCGTGTGA |
| 19[144] | 18[112] | AAGCAAATAAAAAAATAAACAGCCATATTATTTATCCCAATCCAAATA |
| 19[160] | 19[194] | TGTAAACGTTAATATTTTTTTTTTTTTTTTTTTT |
| 2[103] | 7[111] | GGGAGCCCCCACCGAGAGGGCGCGGAAAAACGCTCCCTG |
| 2[135] | 7[135] | TTTGCGTAGAGGATCCAGCATAAACGTCGGTG |
| 2[194] | 2[160] | TTTTTTTTTTTTTTTTCCAGCTGCATTAATGAAT |
| 20[199] | 20[168] | TTTTTTTTTTTTTTTTAAGATTCAAAAGGGTG |
| 20[97] | 19[97] | TTTAGTTAATTTCATCTTAACATAGCGATAGCTTAG |
| 21[112] | 20[128] | AGAAACGATTTTTTGTTTAACGTCATATGATA |
| 21[144] | 16[152] | AAATAATTTTAAATGCTTTTTGTTATTTAAATCTGTAGCC |
| 21[16] | 20[15] | TTTTTTTTTAAGAATAAACACCGGTAAATAAGGCGTTAAATTTTTTTTT |
| 21[160] | 23[194] | AATGCCTAGAAAGGAAGCCTTTATTTCAACGCTTTTTTTTTTTTTTTT |
| 21[168] | 21[199] | GAGTAATGTGTAGGTATTTTTTTTTTTTTTTT |
| 21[40] | 22[13] | AATCATAAGCGTTATACAAATTCTTACTTTTTTTTTTTTTTTT |
| 21[55] | 16[56] | GAAAAAGCCAGTACATATAATTTTCGATGATGA |
| 22[100] | 21[100] | AATAGCAAGCAAATCAGATATGCGTTTTAGCGAACCTCCCGA |
| 22[143] | 27[143] | CTTTACAGAGAGAATAAATTAACTTACATTTCCAACCGATTGAGGGAG |
| 22[194] | 24[160] | TTTTTTTTTTTTTTTTAAGGATAAAAATTTTTAGTGGAAGTGCGAACG |
| 22[71] | 27[71] | TATCCGGTCGTTTTTAAAACCAATCCAGAAGG |
| 23[115] | 20[115] | CGGTTGTACCAAATTCAACCGTTCTA |
| 23[128] | 25[143] | AACATTATGACCCTGTCCATTAGAGAACACCC |
| 23[13] | 24[40] | TTTTTTTTTTTTTTTTCAGTATAAAGCCAACGCTCTTTCCTTA |
| 24[199] | 24[168] | TTTTTTTTTTTTTTTTGTTGATTCCCAATTCT |
| 24[97] | 23[97] | TCCCATCCTAATTTACGATTAACAACGCCAACATGT |
| 25[112] | 22[112] | GCGCATTAGACGGGAGACATAAAAACAGGGAA |
| 25[144] | 20[144] | TGAACAGTACGGTGTCAACCCTCATGCGGGAGCCGGAGACAGTCAAAT |
| 25[16] | 24[15] | TTTTTTTTTATTAAACCAAGTACCTCATTCCAAGAACGGGTTTTTTTTT |
| 25[168] | 25[199] | TTCATTCCATATAACATTTTTTTTTTTTTTTT |

| 25[40] | 26[13] | GCACTCATTATCTTACCGAAGCCCTTTTTTTTTTTTTTTTTTT |
| --- | --- | --- |
| 25[55] | 20[56] | CAAGCAAGCTGTTTAGTGGGCTTAAGGTTTGAA |
| 26[143] | 31[135] | CAGAGGGTAATTGAGCAACCAGACGAAATTATGAAAACGA |
| 26[194] | 26[160] | TTTTTTTTTTTTTTTTAGCTCAACATGTTTTAAA |
| 26[71] | 31[71] | AGAAACAACGACAGAAAGCAAAATCTCAGAAC |
| 27[110] | 29[103] | AGACAAAAGGCAGAGAGATAACCCACAAGAATTTGTAGCGC |
| 27[13] | 28[40] | TTTTTTTTTTTTTTTTTTAAGAAAAGTAAGCAGATTACCATTA |
| 27[160] | 27[194] | TGCTGAATATAATGCTGTTTTTTTTTTTTTTTTT |
| 28[103] | 30[112] | TCACCGACCAGAGCCGCCACAAATGCTTTAAAAATATCG |
| 28[143] | 30[136] | TATTGACGCGGAAGCATTAAGAGG |
| 28[199] | 28[168] | TTTTTTTTTTTTTTTTTCCTTTTGATAAGAGG |
| 28[87] | 25[100] | TTTGGGAAGAAACGCACCCAATAAGTAGGAATCATTACCGCGCCC |
| 29[120] | 24[115] | TCAAAGCGGCTAATATGCGACATTGCAAATGGTCAAT |
| 29[144] | 24[152] | AACTCCAACAGGTCAGTATGCAACAGCTTAATAGTAGATT |
| 29[16] | 28[15] | TTTTTTTTCACCAATGAAACCATCGCAAGGCCGGAAACGTTTTTTTTTT |
| 29[160] | 31[194] | GATTAGATCATTTTGTCTTTACCCTGACTATTTTTTTTTTTTTTTTTT |
| 29[168] | 29[199] | GAGTACCTTTAATTGCTTTTTTTTTTTTTTTT |
| 29[40] | 30[13] | GATAGCAGAAATCACCGGAACCAGAGCTTTTTTTTTTTTTTTT |
| 29[55] | 24[56] | AATCAGTAGTGAAATAGCAAAGTTACAATAATC |
| 29[88] | 27[102] | CGTCAGACGAGTTAAGATAATAACGGAATAC |
| 3[104] | 2[104] | CGCAATGAGTGAGCTCAGGGTGGTTTTAAA |
| 3[13] | 4[40] | TTTTTTTTTTTTTTTTAGCGGGCGCTAGGGCGCTGAATCAGAG |
| 3[160] | 3[194] | GTCGGGAAACCTGTCGTGTTTTTTTTTTTTTTTT |
| 30[111] | 35[103] | CGTTTTCATCGGCATGCTTTTGAGAACCACCAAGGATTA |
| 30[135] | 35[135] | AAGCCCGACGACGATATCGTCATAACGGAACA |
| 30[194] | 32[160] | TTTTTTTTTTTTTTTTATAGTCAGAAGCAAAGCGTGCAAAATTTAGAC |
| 30[71] | 35[71] | CGTTTGCCTGAATTTAAGGAGGTTGAAAGTAT |
| 31[13] | 32[40] | TTTTTTTTTTTTTTTTCACCACCGGAACCGCCTCCATTGGCCT |
| 32[199] | 32[168] | TTTTTTTTTTTTTTTTGGGTAATAGTAAAATG |
| 33[112] | 28[120] | ACCCTCGTTTACCAGAAAGACTTCACAGTTCATCATTAAA |
| 33[144] | 28[152] | AATAGCGAGAGGCTTTGATTGCATAAAATCAGTGCGGATG |
| 33[16] | 32[15] | TTTTTTTTATAAATCCTCATTAAATGATATTCACAAACAATTTTTTTTT |
| 33[168] | 33[199] | GAAGTTTTGCCAGAGGTTTTTTTTTTTTTTTT |
| 33[40] | 34[13] | GCCAGAATGTATAAACAGTTAATGCCCTTTTTTTTTTTTTTTT |
| 33[55] | 28[56] | CGCAGTCTCATCTTTTCCCGCCACCCACCAGTA |
| 33[80] | 28[88] | GTAAGCGTCATACATGTTTCGGTCACCACCCTTTGAGCCA |
| 34[135] | 39[135] | TTACGAGGCGAGAAACACCAGTCAAAGAGGAC |
| 34[194] | 34[160] | TTTTTTTTTTTTTTTTATACCACATTCAACTAAT |
| 34[71] | 39[71] | CAGTGCCTCACCCTCAGCCGTCGAATCTAAAG |
| 35[104] | 34[96] | GGATACGTTAATAAAGAGCAACACTATTACAGGAGTGT |
| 35[13] | 36[40] | TTTTTTTTTTTTTTTTCCTGCCTATTTCGGAACCTTATAGCCC |
| 35[160] | 35[194] | ATCAGTTGAGATTTAGGATTTTTTTTTTTTTTTT |
| 36[199] | 36[168] | TTTTTTTTTTTTTTTTTAATCATTGTGAATTA |
| 37[112] | 32[120] | ATAAGGCTTGCCCTGACATAGTAAACGAACTAAATATTCA |
| 37[144] | 32[152] | GAGTAGTAAATTGGGCGCAGATACAAAGATTCTGGATAGC |
| 37[16] | 36[15] | TTTTTTTTCGTACTCAGGAGGTTTGGAATAGGTGTATCACTTTTTTTTT |

| 37[160] | 39[194] | TTGAGATCCTTATGGCATAGGCTGGCTGACCTTTTTTTTTTTTTTTTT |
| --- | --- | --- |
| 37[168] | 37[199] | GGTTTAATTTCAACTTTTTTTTTTTTTTTTTT |
| 37[40] | 38[13] | AGTACCGCCAAACTACAACGCCTGTAGTTTTTTTTTTTTTTTT |
| 37[55] | 32[56] | CAGAACCGCTGAGTAACTGAAACATGAGGCAGG |
| 37[80] | 32[88] | ACCCTCAGAGCCACCAACTGGTAACTCAAGAGACCAGAGC |
| 37[96] | 38[112] | CCCTCATTGCGGGGTTTAAATGAATTTACCGAACTGACCGCTGCTCA |
| 38[111] | 43[103] | TTCAAGGGATAGCAAGCTTGCTTGTATGGGAAAAGACAG |
| 38[135] | 43[135] | AAATCAACAGCGATTACGAGGCGCCTACAGAG |
| 38[194] | 40[160] | TTTTTTTTTTTTTTTTTCATCAAGAGTAATCTTGCGGAGATCCGCGAC |
| 38[71] | 43[71] | AACACTGACAAAAGGATTCAGCGGCCGCTTTT |
| 39[13] | 40[40] | TTTTTTTTTTTTTTTTCATTCCACAGACAGCCCTCGAACAACT |
| 4[199] | 4[168] | TTTTTTTTTTTTTTTTTGTGAAATTGTTATCC |
| 40[199] | 40[168] | TTTTTTTTTTTTTTTTTAAATTGTGTCGAAAT |
| 41[112] | 36[120] | CTCATCTTTGACCCCCGTAACAAAAACTTTGAGGACGTTG |
| 41[144] | 36[152] | GCGAAACAAAGTACAAACAAGAACGACCAGGCCGATTTTA |
| 41[16] | 40[15] | TTTTTTTTATAATTTTTTCACGTTAAAGGAATTGCGAATATTTTTTTTT |
| 41[168] | 41[199] | TTGTATCATCGCCTGATTTTTTTTTTTTTTTT |
| 41[40] | 42[13] | GAAAATCTCGCCCACGCATAACCGATATTTTTTTTTTTTTTTT |
| 41[48] | 36[56] | CCAAAAAAAAGGCTCGTTTCGTCGCGTAACGGAGGGTTG |
| 41[80] | 36[88] | TTGTATCGGTTTATCAGCCCAATAGACGTTAGTTGCTCAG |
| 42[111] | 40[88] | CTAAAGGTGAATTTCTTAAACAGCAGCAGCGTTTTGCTA |
| 42[135] | 40[120] | AAACGAAAGAGGCAAAAGCAACGGAGACGGTC |
| 42[194] | 42[152] | TTTTTTTTTTTTTTTTAACGGGTAAAATACGTAATGCCACTA |
| 42[71] | 40[56] | GCGCCGACAATGACAAGTTAAAGGAGTGAGAA |
| 43[13] | 42[48] | TTTTTTTTTTTTTTTTTATTCGGTCGCTGAGGCTTGCAGGGACAACCAT |
| 43[152] | 40[152] | ACTTTTTCCTGCTCCA |
| 43[160] | 43[194] | ATGAGGAAGTTTCCATTATTTTTTTTTTTTTTTT |
| 44[150] | 45[119] | TTCGACAATAAACAACATGTTCAGCTAATGCAGAACGCGAGTACCGA |
| 45[120] | 48[112] | CAAAAGGTAAATGCTGATGCAAATAGGTCTGAAGAAACAA |
| 45[67] | 44[67] | AATTTAGGCAGAGAAGAAAAATAATA |
| 46[103] | 55[111] | AAGACAAATAATAAGAGAATATAACCTGTTTATATGTTAG |
| 46[150] | 45[150] | TTTTTTGGGTTATATAACTATATGTAAAGTAATTCTGTCCAGACGATTTT |
| 46[95] | 48[80] | GAACGCGAGAAAACTGAAGAGTCGAATACCA |
| 47[67] | 46[67] | ATTAAGACGCTGATTTTCAAATATAT |
| 48[111] | 50[104] | TAACGGATAAAACAGAAATAAAGAACGGTAATCGTAAAAC |
| 48[127] | 47[150] | ACATCGGGGAGACTACCTTTTTAACCTCCGGCTTAGGTTTTTTTTTTTTT |
| 48[153] | 49[153] | TTGAATATACAGTAACAGAGGTTTAACGTCAGATTT |
| 49[64] | 50[67] | GAAGGGTTAGAACCTACCATATCAATGTACCCCGGTTGATAATCA |
| 49[96] | 47[95] | TTGCACGTTCGCCTGATTGCTTTAATAGTG |
| 5[112] | 0[120] | TACCTCGATAAAGACGTTGGGCGCAACTCACAAGAATAGC |
| 5[144] | 0[152] | CGAGCTCGAATTCGTACGGCCAACGCTTTCCATCCGAAAT |
| 5[16] | 4[15] | TTTTTTTTGGCCGATTAAAGGGATCGGGAGCTAAACAGGATTTTTTTTT |
| 5[160] | 7[194] | ATCATGGGCTCACACGGCTGACGCATTTCACATTTTTTTTTTTTTTTT |
| 5[168] | 5[199] | TCATAGCTGTTTCCTGTTTTTTTTTTTTTTTT |
| 5[40] | 6[12] | TTTAGACAACATCACTTGCCTGAGTAGTTTTTTTTTTTTTTTTT |
| 5[55] | 0[48] | GTACGCCAGACGTGGCGTAGCGGTCCGTGGACTCCAACGT |

| 5[80] | 0[88] | TTTATAATCAGTGAGGCCGATTTAGCCGCGCTCCAGTTTG |
| --- | --- | --- |
| 50[103] | 52[96] | TAGCATGTCTATTTTTGAGAGATCATTAGCAAAATTAAGC |
| 50[145] | 51[145] | TTTTTTTTTTGGAGCAAACAAGAGATCATTGCCTGAGAGTCTTTTTTTTT |
| 51[120] | 48[128] | CTATCAGGATCGATGAAATTGCGTAGATTTTCTACCTTTT |
| 51[67] | 52[67] | GCTGATAAATTAACATAAAGCTAAAT |
| 52[145] | 54[126] | TTTTTTTTTTTTTTTTACATCCAATAAATCATAATAGTAGCGCAAAGACA |
| 53[120] | 51[119] | ATTCTACTACAGGCAAGGCAAAGATACAAAGG |
| 53[67] | 53[87] | AACCTGTTTAGCTATATTTTC |
| 53[96] | 58[89] | GCGCGAGCCACAATCAATAGAAACTCCTTATTTAATT |
| 54[125] | 55[148] | CCACGGGAAAATACATACATAAAGGTGGCAACATT |
| 54[148] | 53[145] | TTTTTTTTTTTTTTTTTATAAAAGAAATAGCATTATTTTTTTTTTTTTTTT |
| 55[112] | 53[119] | CAAACGTAAATAAGTTTATTTTGTTGAAAAGGTGGCATCA |
| 55[64] | 55[79] | CCAAAAGAACTGGCAT |
| 55[96] | 44[88] | TTACGCAGTCAACAATAGATAAG |
| 6[135] | 11[135] | AGTAAACAATTCATGCAGTGACTCGCTGGCGA |
| 6[194] | 8[160] | TTTTTTTTTTTTTTTTTAAATCATTTCTCCGAACTGAATTGCCCGCTT |
| 6[71] | 11[71] | CAATACTTGACCTGAAAAATGGATACCGCCTG |
| 60[125] | 42[112] | TAACAGGATTAGCAGAGCGAGGCATCGGAACGAGGGTAGAATACA |
| 60[157] | 42[144] | TAACAGGATTAGCAGAGCGAGGGATTAAGAATTCATATGGTTTACCAGCGCCAA |
| 60[93] | 42[80] | TAACAGGATTAGCAGAGCGAGGGCGGGATCGTCACCCTCTTGATAC |
| 61[130] | 38[144] | TAACAGGATTAGCAGAGCGAGGTGTTACTTGGTGTACACGGATATT |
| 61[34] | 37[54] | TAACAGGATTAGCAGAGCGAGGTAGAAAGATAGTTAACCAGTACACCCT |
| 61[66] | 38[80] | TAACAGGATTAGCAGAGCGAGGAACAACTTTCTTTCCAGGAACCCA |
| 61[98] | 41[111] | TAACAGGATTAGCAGAGCGAGGAATCATAAGGGATCTTCGAACA |
| 62[157] | 42[136] | TAACAGGATTAGCAGAGCGAGGAGATGAACAGCCGGAATACCAAGCCCAACCTA |
| 62[93] | 42[72] | TAACAGGATTAGCAGAGCGAGGTTTTGTCGTCAACAGTGCCTTTAACGATAGTT |
| 63[130] | 34[144] | TAACAGGATTAGCAGAGCGAGGAGAACTGGACAGGTAGATAACGCC |
| 63[34] | 33[54] | TAACAGGATTAGCAGAGCGAGGATATAAGATTATTCAGTGCCCGGAAAG |
| 63[66] | 34[80] | TAACAGGATTAGCAGAGCGAGGTACCAGGCTGAGACTCTAAGTTTT |
| 63[98] | 37[111] | TAACAGGATTAGCAGAGCGAGGGGAAGAAAAATCTTATTCGTGA |
| 64[157] | 38[136] | TAACAGGATTAGCAGAGCGAGGACATTATTCTCATTATACCAGAACCATTACCC |
| 64[93] | 38[72] | TAACAGGATTAGCAGAGCGAGGTAAGAGGCGGATAAGTGAACCGCCTGTACCGT |
| 65[130] | 30[144] | TAACAGGATTAGCAGAGCGAGGGTCCAATAATAAATCACAAAAAGA |
| 65[34] | 29[54] | TAACAGGATTAGCAGAGCGAGGTCAGACGCTCAGAGATAATCACACCGT |
| 65[66] | 29[87] | TAACAGGATTAGCAGAGCGAGGCGCCGCCATCAGAGCCATAGCCCCGCCTTTAG |
| 65[98] | 33[111] | TAACAGGATTAGCAGAGCGAGGTTGAATCCCCCTCCATGACATA |
| 66[157] | 34[136] | TAACAGGATTAGCAGAGCGAGGGAATGACCCTGCGGAAAAAACCAAAAAAGGAA |
| 66[93] | 34[72] | TAACAGGATTAGCAGAGCGAGGCGCCACCCGCATTGACCCGTTCCAAACGGGGT |
| 67[130] | 26[144] | TAACAGGATTAGCAGAGCGAGGGCTGTAAAGGAAGTAGTAAAAAGT |
| 67[34] | 25[54] | TAACAGGATTAGCAGAGCGAGGGCACCATAGCCGAACAATAGCCGAGAA |
| 67[98] | 29[119] | TAACAGGATTAGCAGAGCGAGGGGTGAATTATCACCGGTTTTAATTCGAGCT |
| 68[93] | 30[72] | TAACAGGATTAGCAGAGCGAGGAAACCGAGTTAGAGCCTCAAGTTTCTTATTAG |
| 69[130] | 22[144] | TAACAGGATTAGCAGAGCGAGGTAGTTTGAAATACTTTTATGCAGC |
| 69[34] | 21[54] | TAACAGGATTAGCAGAGCGAGGGGCTGTCAACAGTAATCATATTTACTA |
| 7[112] | 9[103] | CCATCTGTGCTACGTGGTGCAAGAGTCTGTCAATATTTT |
| 7[12] | 8[40] | TTTTTTTTTTTTTTTTTAAGAACTCAAACTATCGGCATTCACCA |

| 70[93] | 26[72] | TAACAGGATTAGCAGAGCGAGGCGCCATATGCATGTAGTTTTCATCTAAGAGCA |
| --- | --- | --- |
| 71[34] | 17[54] | TAACAGGATTAGCAGAGCGAGGATACCGATATTAATAATCAATTTTTTT |
| 72[157] | 21[143] | TAACAGGATTAGCAGAGCGAGGGATTGTATCACCATCAAAAAATGA |
| 72[93] | 22[72] | TAACAGGATTAGCAGAGCGAGGTCCTTGAACTGACCTAAACGCGAGAGAAGGCT |
| 73[130] | 14[144] | TAACAGGATTAGCAGAGCGAGGAGCTTTCAATGTGAGCTGGGATAG |
| 73[34] | 13[55] | TAACAGGATTAGCAGAGCGAGGAACAAACATTTTGCCTTTGCCTATCTTT |
| 74[125] | 14[112] | TAACAGGATTAGCAGAGCGAGGTTATCAGATGATGGCTCGTAACC |
| 74[157] | 17[143] | TAACAGGATTAGCAGAGCGAGGCCTATTAATCAACGATATTTGCCA |
| 74[93] | 18[72] | TAACAGGATTAGCAGAGCGAGGCAGAAGGATACCTGAGTGCACCCATTAAATCA |
| 75[130] | 10[144] | TAACAGGATTAGCAGAGCGAGGCGCCATTCTGTGCTGCACGACGGC |
| 75[34] | 9[54] | TAACAGGATTAGCAGAGCGAGGATCTGGTGCGGTCAAAAAATAAGATAG |
| 75[66] | 10[80] | TAACAGGATTAGCAGAGCGAGGGCTGAACCCCACGCTGGCGCGAAC |
| 75[98] | 13[111] | TAACAGGATTAGCAGAGCGAGGCGATCGGTGCGGAAATGAATCT |
| 76[157] | 14[136] | TAACAGGATTAGCAGAGCGAGGAAGGGGGAAGGCTGCGTATCGGCCGTCACGTT |
| 76[93] | 14[72] | TAACAGGATTAGCAGAGCGAGGCAACAGTGTCAAATATAATAGATTCAATTCGA |
| 77[130] | 6[144] | TAACAGGATTAGCAGAGCGAGGTTTACGCTTATAGGGGCCTGGTTG |
| 77[34] | 5[54] | TAACAGGATTAGCAGAGCGAGGTTGGCAGCTTGCTGAGTAATAGGAACG |
| 77[66] | 6[80] | TAACAGGATTAGCAGAGCGAGGGACGCTCACCAGCCATAAATTAAC |
| 77[98] | 9[111] | TAACAGGATTAGCAGAGCGAGGCGACAGTGCGGCATGTGAGTTC |
| 78[157] | 10[136] | TAACAGGATTAGCAGAGCGAGGGGCACGAACGCCCTGGGCACGACTCAGTGCCA |
| 78[93] | 10[72] | TAACAGGATTAGCAGAGCGAGGTATTACCGATCGTCTGAGCGTAAGTGATAGCC |
| 79[130] | 2[144] | TAACAGGATTAGCAGAGCGAGGCAACATACCACTGCCCGCGCGGGG |
| 79[34] | 1[54] | TAACAGGATTAGCAGAGCGAGGTCGTTAGGCAAGTGAGAAAGGATCACC |
| 79[66] | 2[80] | TAACAGGATTAGCAGAGCGAGGTTGCTTTGCCACACCCGAGCTTGA |
| 79[98] | 5[111] | TAACAGGATTAGCAGAGCGAGGCCTGGGGTGCCTTACTAATTGT |
| 8[103] | 10[112] | GAAATACCCAGCAAATGAAGCCTCTTCGCTAGCCAGGGT |
| 8[199] | 8[168] | TTTTTTTTTTTTTTTTGTCCCGCCAAAATAAC |
| 80[157] | 6[136] | TAACAGGATTAGCAGAGCGAGGGTTGCGCTGAGCCGGACCGGGTACGTGTAATG |
| 80[93] | 6[72] | TAACAGGATTAGCAGAGCGAGGCGTAACCAACGAGCACGAAGTGTTCGTTGTAG |
| 81[130] | 2[136] | TAACAGGATTAGCAGAGCGAGGCGGCGGCAAAATCCCTTATTGATTGCCAGAGGCGG |
| 81[66] | 0[72] | TAACAGGATTAGCAGAGCGAGGGAACAAGAGTCCACTA |
| 81[98] | 1[111] | TAACAGGATTAGCAGAGCGAGGCCGAGATAGGGTTGACCTTCTT |
| 84[101] | 54[64] | TAACAGGATTAGCAGAGCGAGGGATTAAGAATTCATATGGTTTACCAGCGCCAA |
| 87[66] | 45[95] | TAACAGGATTAGCAGAGCGAGGTCCTGAACGCATTTTCGAGCCAG |
| 9[112] | 4[120] | TTCTAAGTGGTTGTGAGGGCTTAAAAGCAACTGTGTAAAG |
| 9[144] | 4[152] | TAAGTGTCCTTAGTGCTCTGACCTCCTTGAATATTCCACA |
| 9[16] | 8[15] | TTTTTTTTTAAAAGGGACATTCTGGTCACACGACCAGTAATTTTTTTTT |
| 9[168] | 9[199] | TCAACCTTATGACAATTTTTTTTTTTTTTTTT |
| 9[40] | 10[13] | GCCAACAGCCGAACGAACCACCAGCAGTTTTTTTTTTTTTTTT |
| 9[55] | 4[56] | AACCCTTCTCTTTGATTGTAATATCTGCTTTCC |
| 9[80] | 4[88] | AATACGTGGCACAGACCATCACGCTGCAACAGTACTATGG |
| 92[117] | 46[104] | TAACAGGATTAGCAGAGCGAGGAATTTATCAAAATCATCCAATCGC |
| 93[58] | 48[64] | TAACAGGATTAGCAGAGCGAGGAGTTACAAAATCGCGC |
| 98[109] | 49[95] | TAACAGGATTAGCAGAGCGAGGAGGGTAGCAATCATAAATTAT |

**Supplementary Data 1:** Staple list for pin pore, wheel pore and T pore.
